# Trade‐off of different deep learning‐based auto‐segmentation approaches for treatment planning of pediatric craniospinal irradiation autocontouring of OARs for pediatric CSI

**DOI:** 10.1002/mp.17782

**Published:** 2025-04-01

**Authors:** Alana Thibodeau‐Antonacci, Marija Popovic, Ozgur Ates, Chia‐Ho Hua, James Schneider, Sonia Skamene, Carolyn Freeman, Shirin Abbasinejad Enger, James Man Git Tsui

**Affiliations:** ^1^ Medical Physics Unit Department of Oncology McGill University Montreal Quebec Canada; ^2^ Department of Radiation Oncology St. Jude Children's Research Hospital Memphis USA; ^3^ Department of Radiation Oncology Jewish General Hospital Montreal Canada; ^4^ Gerald Bronfman Department of Oncology McGill University Montreal Quebec Canada; ^5^ Lady Davis Institute for Medical Research Jewish General Hospital Montreal Quebec Canada

**Keywords:** auto‐segmentation, contouring, carnio‐spinal irradiation, deep learning, radiation oncology, treatment planning

## Abstract

**Background:**

As auto‐segmentation tools become integral to radiotherapy, more commercial products emerge. However, they may not always suit our needs. One notable example is the use of adult‐trained commercial software for the contouring of organs at risk (OARs) of pediatric patients.

**Purpose:**

This study aimed to compare three auto‐segmentation approaches in the context of pediatric craniospinal irradiation (CSI): commercial, out‐of‐the‐box, and in‐house.

**Methods:**

CT scans from 142 pediatric patients undergoing CSI were obtained from St. Jude Children's Research Hospital (training: 115; validation: 27). A test dataset comprising 16 CT scans was collected from the McGill University Health Centre. All images underwent manual delineation of 18 OARs. LimbusAI v1.7 served as the commercial product, while nnU‐Net was trained for benchmarking. Additionally, a two‐step in‐house approach was pursued where smaller 3D CT scans containing the OAR of interest were first recovered and then used as input to train organ‐specific models. Three variants of the U‐Net architecture were explored: a basic U‐Net, an attention U‐Net, and a 2.5D U‐Net. The dice similarity coefficient (DSC) assessed segmentation accuracy, and the DSC trend with age was investigated (Mann–Kendall test). A radiation oncologist determined the clinical acceptability of all contours using a five‐point Likert scale.

**Results:**

Differences in the contours between the validation and test datasets reflected the distinct institutional standards. The lungs and left kidney displayed an increasing age‐related trend of the DSC values with LimbusAI on the validation and test datasets. LimbusAI contours of the esophagus were often truncated distally and mistaken for the trachea for younger patients, resulting in a DSC score of less than 0.5 on both datasets. Additionally, the kidneys frequently exhibited false negatives, leading to mean DSC values that were up to 0.11 lower on the validation set and 0.07 on the test set compared to the other models. Overall, nnU‐Net achieved good performance for body organs but exhibited difficulty differentiating the laterality of head structures, resulting in a large variation of DSC values with the standard deviation reaching 0.35 for the lenses. All in‐house models generally had similar DSC values when compared against each other and nnU‐Net. Inference time on the test data was between 47–55 min on a Central Processing Unit (CPU) for the in‐house models, while it was 1h 21m with a V100 Graphics Processing Unit (GPU) for nnU‐Net.

**Conclusions:**

LimbusAI could not adapt well to pediatric anatomy for the esophagus and the kidneys. When commercial products do not suit the study population, the nnU‐Net is a viable option but requires adjustments. In resource‐constrained settings, the in‐house model provides an alternative. Implementing an automated segmentation tool requires careful monitoring and quality assurance regardless of the approach.

## 1 INTRODUCTION

Accurate delineation of target volumes and surrounding organs at risk (OARs) is essential for radiotherapy treatment planning to ensure the delivery of precise radiation treatments. Expert annotators must contour multiple organs on every slice on which they appear, which is time‐consuming and prone to inter and intra‐observer variability.^1^ Reviewing the manually segmented contours is also subject to the same challenges, and deviations from QA protocols can negatively impact oncologic outcomes.^2^ Consistent delineation contributes to the robustness of radiation therapy plans. For these reasons, automating contouring and contour verification has the potential to significantly improve efficiency and accuracy.^1^

With the rapid advancements in artificial intelligence (AI), there has been a shift toward deep learning‐based methods.^3^ Various commercial auto‐segmentation software packages for medical images are now available.^4^, ^5^, ^6^, ^7^ LimbusAI is one example of a deep learning‐based segmentation tool developed specifically for radiotherapy purposes.^8^, ^9^, ^10^, ^11^, ^12^, ^13^ It is built upon a variant of the U‐Net architecture, has been validated clinically in the adult population, and received Food and Drug Administration (FDA) approval in September 2020.^14^

However, there are instances where creating in‐house solutions tailored to specific institutional needs proves advantageous. While commercial products and externally validated tools are designed to be generalizable, their performance suffers when the characteristics of the new data significantly diverge from those of the training data. Furthermore, if the organ of interest and/or target is not supported by the model, then training a new model is required.

Auto‐segmentation for pediatric patients undergoing craniospinal irradiation (CSI) illustrates the necessity of customizing auto‐segmentation tools. Commercial products are trained on adult populations and may not perform well for pediatric patients. Moreover, they lack support for organs crucial in pediatric treatment planning, such as the vertebral bodies where heterogeneous doses may impact growing children, potentially leading to kyphosis or scoliosis.

One approach is to adopt out‐of‐the‐box solutions such as the nnU‐Net, a widely recognized deep learning‐based auto‐segmentation tool within the medical community.^15^, ^16^ It allows health professionals with intermediate‐level expertise in AI to train models tailored to their needs, such as automating treatment planning for 3D‐conformal pediatric CSI.^17^ The nnU‐Net has been demonstrated to consistently outperform many other models in international open challenges.^15^, ^16^ The major drawback of this method is that it requires considerable computational resources, such as graphics processing units (GPUs), which could limit its implementation in some institutions.

In‐house models can be a less computationally intensive alternative, with inference performed on a central processing unit (CPU). However, some in‐depth knowledge of AI is necessary. While surveys conducted among healthcare professionals in radiation oncology have shown that a majority of respondents believe that AI will improve patient treatment in the near future, knowledge of AI was moderate.^18^, ^19^, ^20^ In this study, the performance of different segmentation strategies, including commercial, out‐of‐the‐box, and in‐house models, was evaluated and compared on a pediatric population undergoing CSI. The trade‐offs of these different strategies were discussed and the process of constructing in‐house deep learning models was explored.

## 2 METHODS

This retrospective study was conducted in accordance with the applicable standard operating procedures of participating institutions, Institutional Review Boards (IRBs) approval, and data sharing agreement between St. Jude Children's Research Hospital and the McGill University Health Centre (MUHC).

### 2.1 Dataset

CT images obtained with 120 kVp on Philips IQon Spectral CT scanner (Philips Healthcare, Cleveland, OH) for CSI treatment planning at St. Jude Children's Research Hospital between October 2016 and October 2021 were curated, excluding scans without images covering from above the head to the thigh and a patient with severe scoliosis. The final dataset included CT scans from 142 pediatric patients. All scans underwent atlas‐based segmentation of OARs (*n* = 18) followed by manual correction. Prior to transfer to the MUHC, all scans were anonymized. The pixel resolution was consistently 0.98 mm/pixel, with a slice thickness of 1.5 mm/slice. The OARs included: the clinical target volume of the spinal canal, vertebral bodies, brain, brainstem, eyes, lenses, optic nerves, chiasm, cochleae, esophagus, lungs, and kidneys.

Images were randomly split with an 80:20 ratio, resulting in 115 and 27 patients assigned to the training and validation datasets, respectively. Further analysis was conducted to ensure that the data distribution between the training and validation subsets was similar in age and individual OAR size (Figure S1).

The test dataset came from the MUHC and the CT images were obtained with 120 kVp on Philips Brilliance Big Bore. Pediatric patients undergoing CSI between February 2009 and March 2021 were identified. Only the patients (*n* = 16) who had the complete set of 18 OARs delineated were included in the test dataset. The mean pixel resolution was 1.17 mm/pixel (σ: ± 0.16; min: 0.84 – max: 1.37) and the mean slice thickness was 2.88 mm/slice (σ: ± 0.70; min: 2–max: 5).

### 2.2 Commercial model

The DICOM files of the validation and test datasets were provided along with the desired structures for contouring. Subsequently, LimbusAI automatically generated from the raw input images the *RT_Struct* DICOM files containing the delineation of the OARs. No pre‐processing was required and LimbusAI v1.7 was used. This software was chosen for this study as it is the commercial model used at our institution.

### 2.3 nnU‐Net

The nnU‐Net served as the out‐of‐the‐box segmentation tool. It does not support DICOM files as input, so the DICOM files of whole‐body CT scans and their corresponding reference segmentation masks for each OAR were first converted to NIfTI format. Data outside the body contour were set to−1000 HU to remove irrelevant information. Patients were then positioned at the center of the image. The final axial slices measured 512 pixels in width (lateral direction) by 256 pixels in height (anterior‐posterior direction). For overlapping OARs, the labels were made mutually exclusive by cropping the smaller structures from the larger structures they overlapped with. For the test dataset, after conversion to NIfTI format, the images and corresponding reference masks were resized using nearest‐neighbor interpolation.

The default training process of nnU‐Net employed a 5‐fold cross‐validation scheme, with each fold trained for 1000 epochs. The training process was conducted on the computational clusters provided by the Digital Research Alliance of Canada (alliancecan.ca), with 50 GB of RAM, GPU support, and a computation time of 10 days requested. The 2D network was trained. The best configuration for inference was a full resolution cascade model.

### 2.4 In‐House Model

**FIGURE 1:**
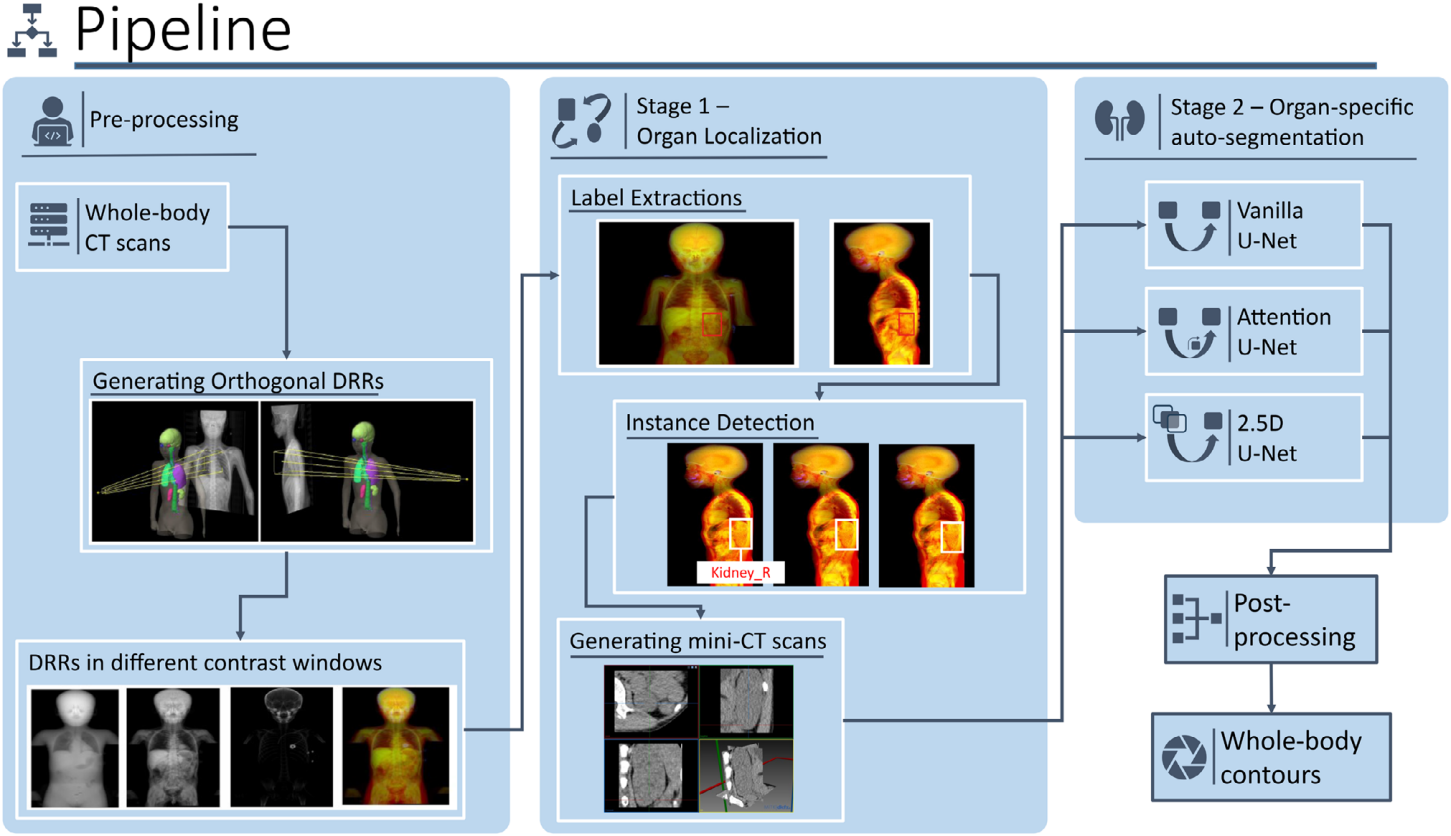
Pipeline of the in‐house model. Pairs of orthogonal DRRs were generated from whole‐body CT scans with four contrast windows. In the organ localization stage, labels containing the coordinates of the structure‐specific ROIs were extracted using YOLOv5 to generate mini‐CT scans. The scans were then fed to the organ‐specific auto‐segmentation stage. Three different U‐Net variants were trained to infer segmentation masks that were then post‐processed and combined to obtain the OARs contours in the whole‐body scans. DRR, digitally reconstructed radiographs;OARs, organs at risk; ROI, region of interest.

We pursued an in‐house approach that was less computationally demanding. To leverage parallel computing of clusters, multiple structure‐specific models were trained in two stages. In the first stage, a region containing the organ of interest was extracted from the whole‐body CT scan, resulting in a smaller 3D‐CT scan. This region of interest (ROI) was then used as input in the second stage, where an organ‐specific model was trained to auto‐segment the organ of interest (Figure 1). The same pre‐processing applied for the nnU‐Net was used to convert DICOM to NIfTI.

#### 2.4.1 Organ localization

Orthogonal digitally reconstructed radiographs (DRRs) were generated for each patient, and organ‐specific instance detection algorithms (YOLOv5) were trained to identify the bounding boxes containing the organs of interest in the resulting 2D images (Figure 1—see *Instance Detection* box). YOLO, a widely used real‐time object detection algorithm, was chosen for its speed and efficiency.^21^ In brief, the model divides the input image into a grid and, for each grid cell, predicts the probability of an object's presence as well as its bounding box coordinates. It processes the whole image in a single pass using a convolution neural network. For this study, two YOLO models were trained for each organ, one for each DRR. The 3D ROIs were determined by integrating the coordinates of the inferred orthogonal bounding boxes.

To improve contrast and allow better visualization of certain organs, the HU values of whole‐body CT scans were first clipped within a specific range. Three HU contrast windows were determined: [−1500, 0], [0, 50], and [250, 3000] for lung, soft tissue, and bone, respectively. DRRs were then generated by taking the sum along an axis and normalizing the resulting images to a range between 0–255.

**FIGURE 2:**
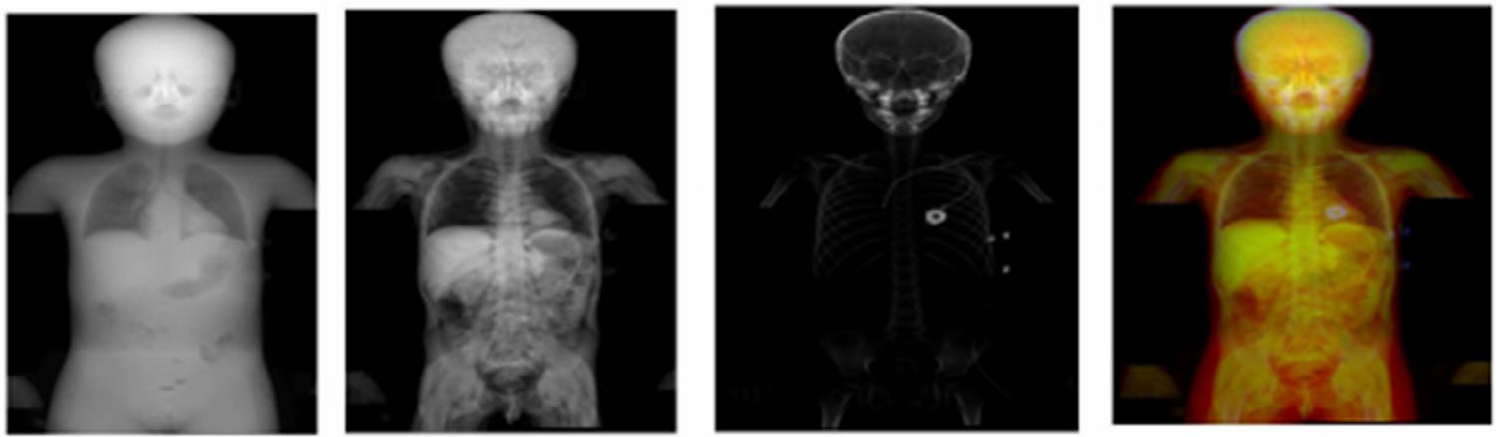
The DRRs in the coronal plane were generated with different contrast windows. The far‐left image shows the DRR when the HU of the pixels was clipped from −1500 to 0, which created a specific lung contrast window suitable for air detection. The middle‐left image displays the DRR for a soft tissue window, where the HU pixel values were clipped between 0 and 50. The middle‐right image shows the DRR for a bone window, achieved by clipping the HU from 250 to 3000. The far‐right shows the composite window DRR. Note, this patient had a cardiac medical device. DRR, digitally reconstructed radiographs.

To accommodate YOLOv5's requirement for a 3‐channel input, each image was duplicated and concatenated, resulting in a 3‐channel image. Additionally, a composite window was also generated by concatenating DRRs from the lung, soft tissue, and bone windows into one image with 3 corresponding channels (Figure 2). Each model was trained on all four contrast windows, and the optimal HU windowing was determined empirically by comparing generated bounding boxes to the reference in the validation set (Table S1).

##### 2.4.1.1 Training to localize body structure ROIs

**FIGURE 3:**
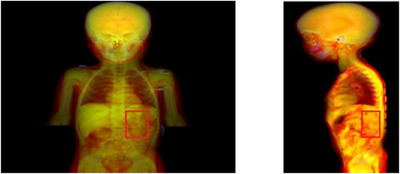
Left shows the DRR in the composite window for the coronal plane with the bounding box (red rectangle) encompassing the left kidney. The sagittal slices within this bounding box were used to reconstruct the DRR in the sagittal plane. DRR, digitally reconstructed radiographs.

For body structures, the training labels consisted of bounding boxes that encompassed the segmentation masks of the organs of interest in the coronal and sagittal planes (Figure 3). The coronal DRRs were obtained through the summation of pixel values in the anterior‐posterior dimension. For sagittal images, only the slices within the lateral extent of the organ of interest were selected to avoid irrelevant lateral information outside the bounding box. Two YOLO models were then trained, one for the coronal plane and another for the sagittal plane. The training was done with a batch size of 32 and a maximum number of epochs of 600.

##### 2.4.1.2 Inference of the body structure ROIs

The inference involved a two‐step process. For each organ, YOLO initially predicted the bounding box on the coronal DRR in the optimal contrast window. Following this, a sagittal DRR was constructed by summing the sagittal slices within the lateral extent of the coronal bounding box obtained in the first step. Subsequently, YOLO predicted the bounding box of the organ of interest on the newly generated sagittal DRR in the optimal contrast window. The resulting 3D ROI was determined by integrating the lateral coordinates of the bounding box inferred from the coronal image with the superior‐inferior and anterior‐posterior coordinates inferred from the sagittal image. Extra pixels were added around the predicted bounding boxes in the axial plane to add contextual information for the subsequent organ segmentation stage (+20 pixels for the lungs; +15 pixels for the CTV spine, OTV VB, kidneys, and esophagus; +10 pixels for the brain and brainstem).

##### 2.4.1.3 Training to localize the head structure ROIs

A different approach was employed for the smaller head structures, which included the chiasm, cochleae, eyes, lenses, and optic nerves. The height and width of the reference bounding boxes for the head structures were increased by a factor of two to provide additional contextual information and facilitate the detection of smaller structures.

Following a similar approach used for body structures, we generated the reference labels, but in the sagittal and axial orientations. First, the inferior border of the brainstem was identified to locate the head area. Then, two sets of sagittal DRRs were generated by taking the sum of sagittal slices of the head from left to the midline and from the midline to right. Second, axial DRRs were created by first locating the superior‐inferior extent of the organs of interest and then taking the sum along the cranial‐caudal dimension. Only the bone window was used for the head structures as it was found to be the most effective in identifying these structures. Two YOLO models were trained, one for the sagittal plane and another for the axial plane, to detect the location of the head OARs.

##### 2.4.1.4 Inference of the head structures ROIs

During the inference on the validation and test datasets, the sagittal YOLO model was used to first find the bounding boxes’ coordinates in the sagittal planes. The axial DRRs were then generated by summing the axial slices within the ROIs found with the sagittal YOLO. The 3D ROIs were then extracted by combining the bounding boxes’ superior‐inferior extent in the sagittal plane with the anterior‐posterior and lateral extent coordinates in the axial plane.

#### 2.4.2 Organ‐specific auto‐segmentation

Organ‐specific 3D scans were created in NIfTI file format based on the 3D bounding boxes generated in the previous step. Padding of 0 was used to obtain a uniform size for the input image. The maximum size for each OAR is listed in Table S2. We explored three variants of the U‐Net architecture.

First, a basic U‐Net was implemented as proposed by Ronneberger et al.^22^ Our implementation of the U‐Net differed slightly from the original architecture. Details of the model architecture can be found in the Supplemental Material (see also Figure S3). The second model we explored was an attention U‐Net model, which has previously demonstrated improved performance compared to the basic U‐Net in medical image classification tasks (Figure S4).^23^ Finally, we also investigated the 2.5D U‐Net, which was based on the basic U‐Net but required a three‐channel input (see Figure S5).^24^ These three channels corresponded to the target axial slice, sandwiched between the superior and inferior axial slices. The model's output was the segmentation mask specifically for the middle axial slice.

For the body organs, HU values were clipped to a range defined by the lowest value between the 10th percentile HU values inside the segmentation mask and the 10th percentile within a 2 mm rim around the reference segmentation mask, and the highest value between the 90th percentile inside the segmentation mask and the 90th percentile within the 2 mm rim. For the smaller structures in the brain, it was found qualitatively that clipping the HU values between 0 and 250 worked the best. Subsequently, the input HU values were normalized between 0–1. Rotations of ± 15 degrees and ± 15% scaling were performed as a data augmentation technique with a 50% probability to reflect the variations observed in real data.

For all three variants of the in‐house models, the output passed through a sigmoid function to produce a pixel‐wise probability map. A threshold of 0.5 was used for binary classification. In post‐processing, a 3D connected‐components algorithm with a connectivity of 26 was used to extract the single largest component from the predicted segmentation masks.

### 2.5 Evaluation metrics

The Dice Similarity Coefficient (DSC) was used to evaluate the predicted segmentation accuracy:

$$DSC=\frac{2\left(A\cap B\right)+\varepsilon }{\left(A+B\right)+\varepsilon }$$

where ε was a smoothing parameter set to 1e^−7^ to prevent the DSC from going to infinity when the denominator is 0. In this case, the DSC quantified the overlap between the ground truth mask (A) and the predicted mask (B). The 3D DSC was reported in this study.

The contours were then qualitatively reviewed by a radiation oncologist to determine clinical acceptability. The physician used a five‐point Likert scale to score the images (Table 1) inspired by Baroudi et al.^25^

**TABLE 1:** Five‐point likert scale used by radiation‐oncologist to determine the clinical acceptability of automatically‐generated contours.

	Likert scale	Explanation
**5**	Strongly agree	No edits necessary, that is, clinically acceptable as is.
**4**	Agree	Minor edits necessary on several slices.
**3**	Neither agree nor disagree	Major edits necessary, the contours can be salvaged.
**2**	Disagree	Major edits necessary, the user would prefer to start over.
**1**	Strongly disagree	Completely unusable. The contour is entirely incorrect, or more than half of it is truncated.

### 2.6 Statistical analysis

The Kruskal–Wallis H‐test was performed to determine statistically significant differences of DSC values between models for every contoured structure. The Holm‐Bonferroni method was used to adjust for multiple testing with a target alpha level of 0.05. If significant differences were found for an OAR, Dunn's multiple comparison test was used post hoc to identify specific model differences.

Additionally, the Mann‐Kendall Trend test from the *pyMannKendall* package in python^26^ assessed DSC trends with patient age for every model and OAR. The False Discovery Rate (FDR) approach with a target alpha level of 0.05 was used to correct for multiple comparisons. If a trend was observed, the DSC was split into two groups according to patient age (< 10 and ≥10 years). The Mann–Whitney U‐test was then performed to compare their means. A *p*‐value smaller than 0.05 was considered significant.

Finally, results from the validation and external test datasets were compared using the Mann–Whitney U‐test with FDR control for every model and OAR. Once again, a *p*‐value of 0.05 or less indicated statistical significance.

## 3 RESULTS

**FIGURE 4:**
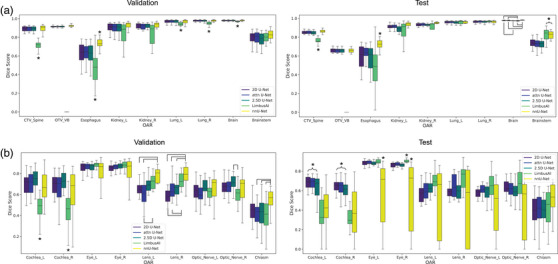
Comparison of the DSC for (a) body and (b) head organs of the validation and test datasets. Asterisks signify that a model's performance was significantly different from all other models, whereas brackets indicate significant differences between a pair of models. DSC, dice similarity coefficient

Table 2 shows the age distribution for all datasets. No significant difference was observed between the training and validation datasets. However, statistically significant differences in the age distribution between the training and test datasets and between the validation and test datasets were found (*p*‐value < 0.01 in both cases). The mean DSC values between all models for every structure on the validation and test datasets were compared (Figure 4; Table 3).

**TABLE 2:** Distribution of patient ages in the training, validation, and test datasets.

Dataset	Number of patients	Age
Training	115	12.3 [9.0–16.8]
Validation	27	14.9 [10.3–17.3]
Test	16	8.5 [6.0–11.0]

*Note*: Age is presented as median [interquartile range].

**TABLE 3:** DSC on the validation and test datasets (mean ± std).

	Validation (*n* = 27)						Test (*n* = 16)					
	2D U‐Net	attn U‐Net	2.5D U‐Net	LimbusAI	nnU‐Net	P_Holm‐Bonferroni_	2D U‐Net	attn U‐Net	2.5D U‐Net	LimbusAI	nnU‐Net	P_Holm‐Bonferroni_
CTV Spine	0.89 ± 0.03	0.89 ± 0.03	0.89 ± 0.03	0.70 ± 0.07	0.90 ± 0.03	**< 0.01**	0.85 ± 0.03	0.85 ± 0.03	0.84 ± 0.03	0.76 ± 0.05	0.86 ± 0.02	**< 0.01**
OTV VB	0.91 ± 0.03	0.91 ± 0.03	0.91 ± 0.03	NA	0.92 ± 0.03	0.13	0.66 ± 0.04	0.66 ± 0.04	0.66 ± 0.03	NA	0.66 ± 0.03	0.92
Esophagus	0.63 ± 0.11	0.63 ± 0.10	0.62 ± 0.11	0.47 ± 0.18	0.73 ± 0.06	**< 0.01**	0.58 ± 0.13	0.63 ± 0.08	0.59 ± 0.13	0.48 ± 0.24	0.71 ± 0.08	**< 0.01**
Kidney L	0.90 ± 0.06	0.90 ± 0.06	0.90 ± 0.06	0.81 ± 0.20	0.92 ± 0.06	0.18	0.91 ± 0.05	0.91 ± 0.05	0.88 ± 0.05	0.86 ± 0.12	0.93 ± 0.04	0.12
Kidney R	0.90 ± 0.08	0.90 ± 0.08	0.90 ± 0.08	0.81 ± 0.19	0.92 ± 0.08	0.24	0.91 ± 0.05	0.91 ± 0.05	0.92 ± 0.05	0.88 ± 0.11	0.94 ± 0.03	0.14
Lung L	0.97 ± 0.02	0.97 ± 0.02	0.97 ± 0.02	0.94 ± 0.03	0.96 ± 0.05	**< 0.01**	0.96 ± 0.02	0.96 ± 0.02	0.95 ± 0.02	0.96 ± 0.02	0.95 ± 0.03	1
Lung R	0.98 ± 0.01	0.98 ± 0.01	0.98 ± 0.01	0.95 ± 0.02	0.98 ± 0.01	**< 0.01**	0.96 ± 0.02	0.96 ± 0.02	0.96 ± 0.02	0.97 ± 0.01	0.96 ± 0.02	1
Brain	0.98 ± 0.01	0.98 ± 0.01	0.98 ± 0.01	0.96 ± 0.02	0.98 ± 0.01	**< 0.01**	0.98 ± 0.01	0.98 ± 0.01	0.97 ± 0.01	0.97 ± 0.02	0.98 ± 0.01	**< 0.01**
Brainstem	0.80 ± 0.06	0.80 ± 0.06	0.78 ± 0.07	0.77 ± 0.10	0.81 ± 0.07	2.35	0.74 ± 0.05	0.73 ± 0.06	0.72 ± 0.04	0.80 ± 0.11	0.82 ± 0.04	**< 0.01**
Cochlea L	0.70 ± 0.10	0.70 ± 0.11	0.75 ± 0.09	0.47 ± 0.17	0.61 ± 0.24	**< 0.01**	0.66 ± 0.11	0.66 ± 0.11	0.65 ± 0.10	0.37 ± 0.22	0.42 ± 0.24	**< 0.01**
Cochlea R	0.69 ± 0.12	0.68 ± 0.12	0.72 ± 0.11	0.45 ± 0.17	0.61 ± 0.24	**< 0.01**	0.63 ± 0.10	0.63 ± 0.10	0.60 ± 0.11	0.32 ± 0.09	0.36 ± 0.26	**< 0.01**
Eye L	0.84 ± 0.13	0.84 ± 0.16	0.84 ± 0.14	0.88 ± 0.04	0.76 ± 0.24	2.56	0.86 ± 0.10	0.84 ± 0.19	0.84 ± 0.14	0.90 ± 0.03	0.54 ± 0.33	**< 0.01**
Eye R	0.82 ± 0.17	0.83 ± 0.17	0.84 ± 0.17	0.88 ± 0.04	0.79 ± 0.18	2.50	0.81 ± 0.21	0.81 ± 0.21	0.81 ± 0.21	0.90 ± 0.02	0.54 ± 0.34	**< 0.01**
Lens L	0.63 ± 0.13	0.60 ± 0.15	0.66 ± 0.15	0.68 ± 0.16	0.70 ± 0.26	**< 0.01**	0.55 ± 0.15	0.57 ± 0.15	0.59 ± 0.17	0.70 ± 0.09	0.49 ± 0.34	0.22
Lens R	0.56 ± 0.14	0.61 ± 0.16	0.63 ± 0.15	0.69 ± 0.16	0.70 ± 0.27	**< 0.01**	0.56 ± 0.16	0.62 ± 0.19	0.57 ± 0.17	0.74 ± 0.12	0.45 ± 0.35	0.12
Optic Nerve L	0.62 ± 0.10	0.60 ± 0.10	0.66 ± 0.11	0.59 ± 0.17	0.60 ± 0.24	0.27	0.56 ± 0.09	0.60 ± 0.08	0.57 ± 0.11	0.66 ± 0.13	0.43 ± 0.29	0.45
Optic Nerve R	0.65 ± 0.13	0.66 ± 0.12	0.69 ± 0.15	0.57 ± 0.17	0.61 ± 0.23	**0.02**	0.61 ± 0.14	0.62 ± 0.09	0.59 ± 0.10	0.68 ± 0.14	0.42 ± 0.30	0.74
Chiasm	0.45 ± 0.12	0.42 ± 0.12	0.44 ± 0.14	0.40 ± 0.16	0.53 ± 0.13	**0.02**	0.40 ± 0.16	0.41 ± 0.17	0.40 ± 0.12	0.42 ± 0.17	0.52 ± 0.16	0.89

*Note*: Significant values are in bold.

Abbreviation: DSC, dice similarity coefficient.

There were some notable differences in the contours between the validation and test datasets, reflecting the different institutional contouring standards. Notably, St. Jude Children's Research Hospital segmented only the vertebral body, while the MUHC also included the vertebral arch, transverse and spinous processes. This difference in segmentation explains the decrease in DSC scores observed for both the in‐house models and the nnU‐Net on the test dataset for OTV VB.

On the validation dataset, post‐hoc tests revealed that LimbusAI had significantly lower mean DSC values than nnU‐Net and all in‐house models for the CTV spine, the brain, the esophagus, the lungs, and the cochleae. Furthermore, nnU‐Net had a significantly higher mean DSC than all other models for the esophagus across both datasets.

On the test dataset, LimbusAI and nnU‐Net demonstrated significantly higher mean DSC values than all in‐house models for the brainstem. Although there were some statistically significant differences for the brain contour, these were not considered clinically relevant as the maximum difference in mean DSC values was 0.01. Additionally, the in‐house models had higher mean DSC values than LimbusAI and nnU‐Net for the cochleae. Finally, nnU‐Net obtained significantly lower mean DSC values than all other models for the eyes, while LimbusAI demonstrated a significantly higher mean DSC value than the other models for the right eye.

### 3.1 Commercial model

The segmentation of vertebral bodies was not supported by LimbusAI and, as a result, was not evaluated. LimbusAI supported the contouring of the spinal canal, but it did not extend beyond the spinal cord's border at ∼L2‐3 nor included the foramina. Therefore, it did not delineate the entire structure containing cerebral spinal fluid, which is the target in CSI (Figure S2).

**FIGURE 5:**
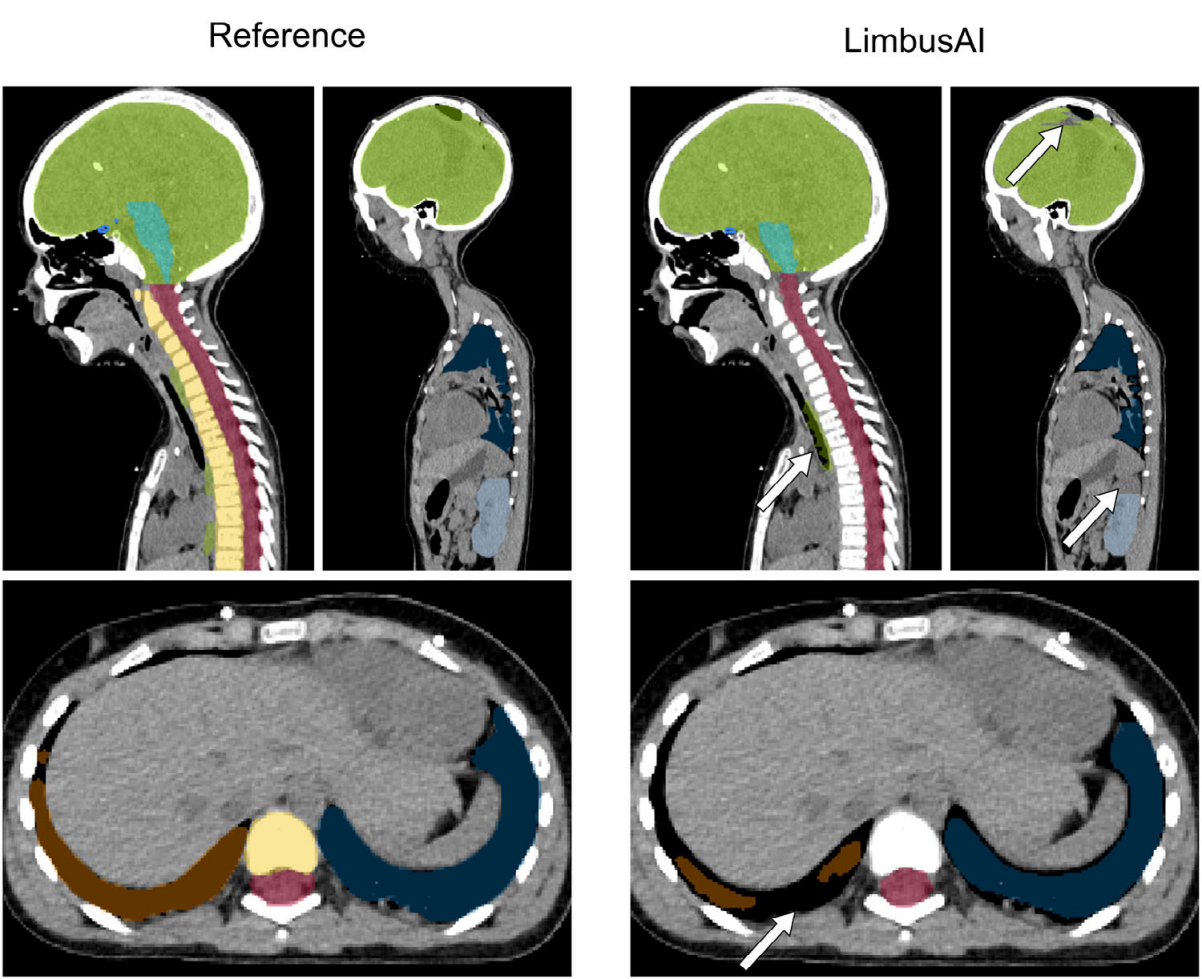
Example of reference contours and contours generated by LimbusAI for a patient on the validation dataset. Contouring issues are highlighted by white arrows. Green: esophagus and brain; turquoise: brainstem; light blue: left kidney; dark blue: left lung; orange: right lung; red: CTV spine; yellow: OTV VB.

A comparison between the OAR contours of LimbusAI and the reference is shown in Figure 5. LimbusAI included the trachea within the esophagus contour across multiple slices and generally exhibited false negatives in its most inferior portion, truncating the esophagus distally. Additionally, the lower and upper regions of the lungs were often not fully contoured. Multiple instances of incomplete contouring of the kidneys were also noticed. This resulted in lower mean DCS values of up to 0.11 on the validation set and 0.07 on the test set, though the differences were not statistically significant. Instances where the brain was not fully contoured were also noticed, as shown in Figure 5.

While LimbusAI accurately identified the location of the cochleae, the segmented structures were often smaller than the reference. On the validation dataset, LimbusAI had significantly higher mean DSC values for the lenses compared to the in‐house basic U‐Net (right lens only) and attention U‐Net (both lenses), but a significantly lower mean DSC value than the 2.5D U‐Net for the right optic nerve. Performance against nnU‐Net is discussed later. On the test dataset, LimbusAI had higher mean DSC values for the lenses and the optic nerves, though the differences were not found to be statistically significant.

**FIGURE 6:**
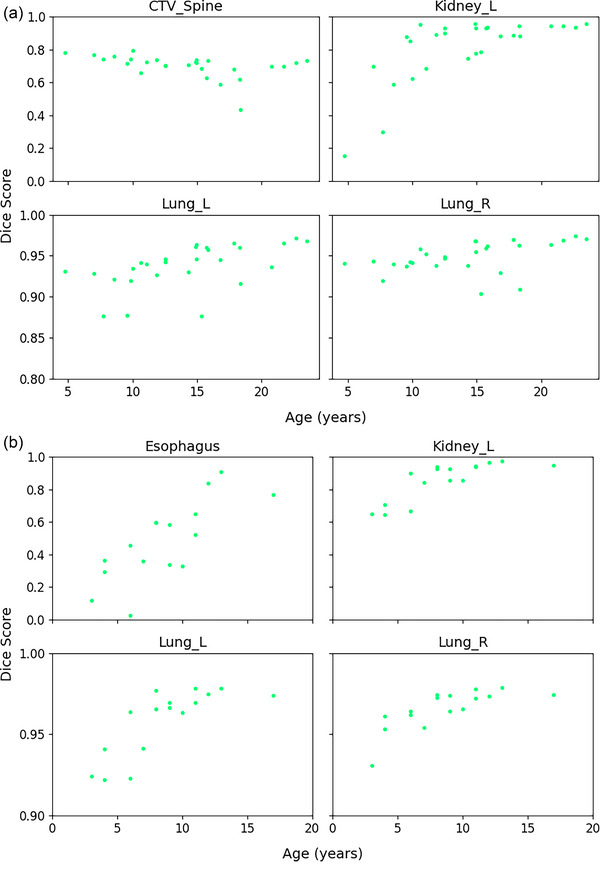
Distribution of DSC values obtained with LimbusAI as a function of patient age for the OARs that exhibited statistically significant trends with age on (a) the validation set and (b) the test set. DSC, dice similarity coefficient; OARs, organs at risk.

The DSC values decreased with age for the spine (*τ* = ‐0.44, *p*‐value = 0.05) and increased for the left lung (*τ* = 0.51, *p*‐value = 0.01), right lung (*τ* = 0.43, *p*‐value = 0.04) and left kidney (*τ* = 0.53, *p*‐value = 0.01) on the validation dataset (Figure 6a). On the test dataset, increasing trends were observed for the esophagus (*τ* = 0.60, *p*‐value < 0.01), left lung (*τ* = 0.68, *p*‐value < 0.01), right lung (*τ* = 0.70, *p*‐value < 0.01), and left kidney (*τ* = 0.75, *p*‐value < 0.01) (Figure 6b). We further split the patients in two age groups (< 10 and ≥10 years) and found significant differences between groups (see Tables 3 and 4).

**TABLE 4:** DSC (mean ± std) of pediatric patients obtained with LimbusAI on the validation dataset for the OARs that exhibited a statistically significant trend with age.

	< 10 years (n = 6)	≥ 10 years (n = 21)	*p*‐value
CTV Spine	0.75 ± 0.02	0.69 ± 0.07	**< 0.01**
Kidney L	0.58 ± 0.27	0.88 ± 0.09	**< 0.01**
Lung L	0.91 ± 0.02	0.95 ± 0.02	**< 0.01**
Lung R	0.94 ± 0.01	0.95 ± 0.02	**0.04**

Abbreviations: DSC, dice similarity coefficient; OARs, organs at risk.

**TABLE 5:** DSC (mean ± std) of pediatric patients obtained with LimbusAI on the test dataset for the OARs that exhibited a statistically significant trend with age.

	< 10 years (n = 10)	≥ 10 years (n = 6)	*p‐*value
Esophagus	0.37 ± 0.19	0.67 ± 0.20	**0.03**
Kidney L	0.81 ± 0.12	0.94 ± 0.04	**< 0.01**
Lung L	0.95 ± 0.02	0.97 ± 0.01	**0.02**
Lung R	0.96 ± 0.01	0.97 ± 0.00	**0.02**

Abbreviations: DSC, dice similarity coefficient; OARs, organs at risk.

### 3.2 nnU‐Net

The esophagus delineated by the nnU‐Net exhibited a significantly higher mean DSC value compared to the other models for both datasets. On the validation dataset, the nnU‐Net had higher mean DSC values for the lenses (compared to the basic and attention U‐Net for the left lens, and all in‐house models for the right lens) and optic chiasm (compared to LimbusAI, 2.5D and attention U‐Net) (Figure 4a). However, these trends were not observed on the test dataset. Instead, the nnU‐Net demonstrated very large DSC variations for all the small head structures (Figure 4b).

**FIGURE 7:**
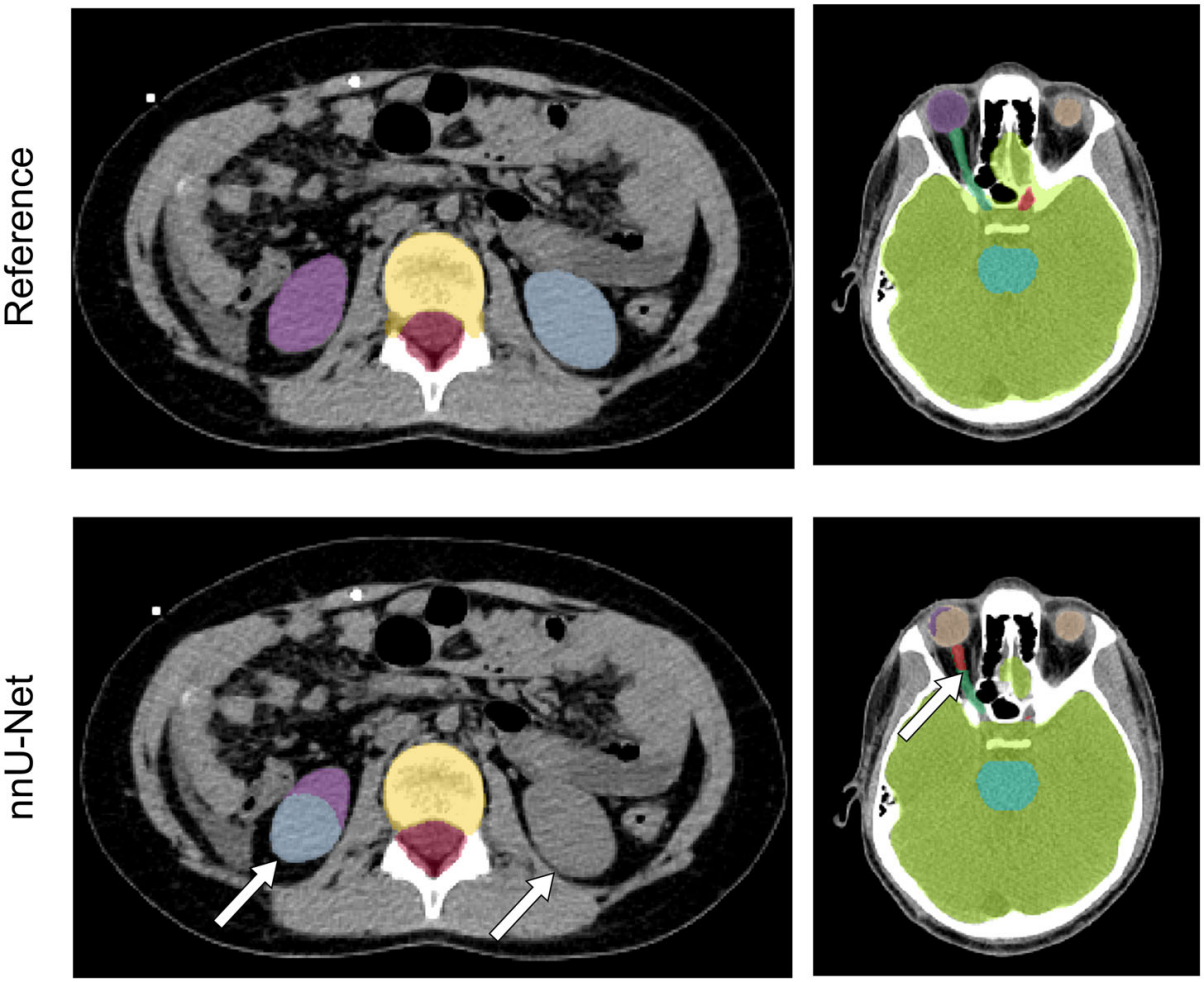
Example of reference contours and contours generated by nnU‐Net for example patients on the validation dataset. Contouring issues are highlighted by white arrows. Green: brain; turquoise: brainstem; light blue: left kidney; purple: right kidney and right eye; beige: left eye; dark green: right optic nerve; red: left optic nerve and CTV spine; yellow: OTV VB.

The performance of the nnU‐Net is shown in Figure 7. The high variation in DSC values for the head structures was attributed to the model's challenge in distinguishing laterality. Of note, cases where the model mistook laterality for other organs such as the kidneys were also noticed, although this seldom happened and did not contribute to noticeable differences in the mean dice score (See Table 5).

### 3.3 In‐House models

**FIGURE 8:**
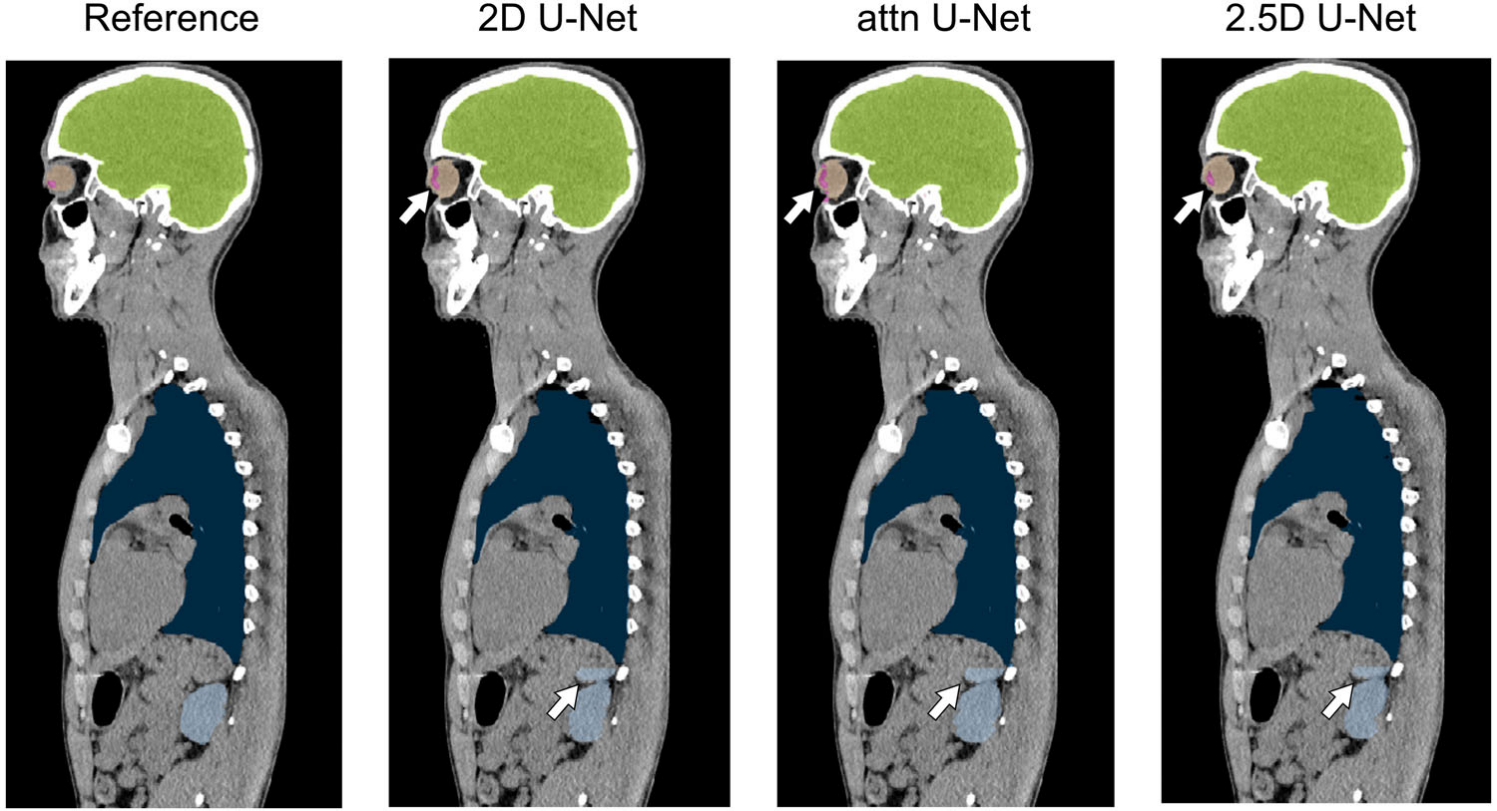
Example of reference contours and contours generated by all in‐house models for an example patient on the validation dataset. Contouring issues are highlighted by white arrows. Green: brain; light blue: left kidney; dark blue: left lung; beige: left eye; pink: left lens.

All in‐house models had similar mean DSC values when compared against each other. For the body structures, the DSC values for all in‐house models were comparable to nnU‐Net apart from the esophagus on both datasets. Additionally, the mean DSC values for the brainstem on the test dataset were statistically significantly lower when compared to nnU‐Net and LimbusAI. Although incidences of false positives and/or false negatives were observed for the kidneys, this did not translate to a statistically significant difference in the mean DSC values. For the head structures, there was less variability in the DSC values for the cochleae and eyes when compared to nnU‐Net, although the performance was only statistically significant for the test dataset. The in‐house models exhibited more false positives and statistically significant differences for the lenses on the validation dataset (Figure 8).

### 3.4 Clinical acceptability

Table 6 summarizes the qualitative evaluation results for each organ contour across all auto‐segmentation models. The percentage of contours in the validation and test sets with a score of 4 or higher was reported, as this was the threshold of clinical acceptability. A full breakdown of Likert scores is presented in the Supplemental Material (see Figure 6 and 7). Because LimbusAI did not support the segmentation of vertebral bodies, it was given a score of 1 for every patient for this structure. The CTV spine was given a score of 2 because LimbusAI truncated the spinal canal around L2‐3 and did not include the foramina, which can include CSF. With nnU‐Net, 96% and 94% of esophagus contours received a score of 4 or higher on the validation and test sets, respectively. This performance was significantly better than that of the other models, which is consistent with the DSC values. For all models except nnU‐Net, the right kidney was evaluated as clinically acceptable more often than the left kidney despite having minimal differences in DSC scores. Although the in‐house models demonstrated lower clinical performance for the esophagus and kidneys compared to nnU‐Net, they were still found to be superior to initiating the contouring process from scratch. For head organs, nnU‐Net underperformed relative to the other models, with the difference being greater on the test set.

**TABLE 6:** Percentage of contours that were given a score greater than or equal to 4 on the five‐point Likert‐scale for every structure and auto‐segmentation model across both the validation and test datasets.

Validation (*n* = 27)						Test (*n* = 16)				
	2D U‐Net	attn U‐Net	2.5D U‐Net	LimbusAI	nnU‐Net	2D U‐Net	attn U‐Net	2.5D U‐Net	LimbusAI	nnU‐Net
CTV Spine	100%	100%	100%	0%	100%	100%	100%	100%	0%	100%
OTV VB	100%	100%	100%	0%	100%	94%	94%	94%	0%	100%
Esophagus	63%	63%	59%	19%	96%	69%	88%	63%	13%	94%
Kidney L	52%	52%	52%	56%	89%	31%	38%	38%	50%	75%
Kidney R	81%	78%	81%	63%	85%	63%	81%	56%	56%	81%
Lung L	100%	100%	100%	100%	96%	100%	100%	100%	100%	100%
Lung R	100%	100%	100%	100%	100%	100%	100%	100%	100%	100%
Brain	100%	96%	100%	85%	100%	100%	100%	100%	100%	100%
Brainstem	100%	100%	100%	93%	100%	100%	100%	100%	100%	100%
Cochlea L	100%	100%	100%	93%	63%	100%	100%	100%	94%	56%
Cochlea R	100%	100%	100%	93%	67%	100%	100%	100%	100%	56%
Eye L	96%	96%	96%	96%	63%	94%	94%	94%	100%	38%
Eye R	96%	93%	93%	96%	67%	94%	94%	94%	100%	38%
Lens L	96%	93%	93%	96%	74%	94%	94%	94%	100%	38%
Lens R	96%	96%	96%	96%	78%	94%	94%	94%	100%	38%
Optic Nerve L	100%	100%	100%	93%	78%	100%	100%	100%	100%	38%
Optic Nerve R	100%	100%	100%	93%	78%	100%	100%	100%	100%	38%
Chiasm	100%	100%	100%	93%	100%	100%	88%	100%	100%	100%

## 4 DISCUSSION

In this study, we evaluated the contours of OARs for pediatric CSI patients generated by LimbusAI and compared them to those from nnU‐Net and three variants of an in‐house deep learning‐based framework. The ease of implementation and performance of different strategies are discussed.

### 4.1 Commercial Model

Firstly, it is important to acknowledge that LimbusAI was trained on adult data, while our dataset comprised pediatric CT images, resulting in different data distributions. Given this discrepancy, we did not anticipate strong performance, particularly for structures prone to age‐related differences including the spinal cord and the esophagus. Additionally, the fat composition around internal organs such as the kidneys changes with age which can affect their detection by auto‐segmentation models. A previous study found that AI models trained on adult data underperformed on pediatric images, especially for patients younger than 2 years old.^27^ However, commercial products still hold value, especially for structures like the smaller head organs that remain relatively consistent across age groups. They can serve as valuable aids in the contouring workflow. A study by Hanna et al. evaluated the performance of Contour ProtégéAI (MIM Software Inc., Cleveland, OH) in the case of pediatric CSI, reported an average DSC score of 0.73 ± 0.04 across all organs.^28^ In comparison, LimbusAI achieved mean DSC scores of 0.71 ± 0.24 and 0.73 ± 0.25 on our validation and test datasets, respectively, for the same OARs.

LimbusAI performed poorly for the esophagus, often including the trachea on multiple slices, as shown in Figure 5. This discrepancy may be attributed to the similarity in appearance between the smaller trachea of children and the esophagus of adults, the population on which the model had been trained. Furthermore, we observed that the distal esophagus was often not segmented. Similarly, the contours of both kidneys were frequently truncated (Figure 5). These contours were considered clinically unacceptable by a radiation oncologist and required major revision or complete rework.

The specific architectural details of LimbusAI were not disclosed; however, we know that the model is based on a variant of the U‐Net.^8^, ^10^ It is plausible that LimbusAI employs a two‐stage approach, wherein an initial organ detection algorithm might be used in the first stage to identify the slices containing the organ of interest. This might then be followed by the segmentation of the organ on axial slices. Underestimating the superior‐inferior extent of the organ might explain the incomplete segmentation of the entire organ.

Another possible explanation could be related to a post‐processing issue. A common post‐processing technique involves retaining only the largest segmented object to eliminate false‐positive floating pixels. If LimbusAI fails to recognize an organ on an axial slice, it could result in a segmentation mask being interrupted at one slice, leading to two separate instances. Subsequently, applying post‐processing to retain the largest object may result in the organ's truncation.

In instances where brain tumors were removed, gross errors in LimbusAI's contours of the brain were observed, despite maintaining a relatively high DSC. The brain DSC value for the patient in Figure 5 was 0.96. This highlights that relying on DSC score on large organs may not always be appropriate. Conversely, the DSC values for the smaller head structures were consistently lower across all models. This primarily reflects a limitation of the DSC metric, which penalizes more small structures for contouring errors.^29^ Although the DSC for the cochleae was statistically significantly lower compared to the other models, the positioning was accurate. The lower DSC is attributed to the segmented mask being smaller than the reference. However, this likely has very little clinical significance and could still be considered clinically acceptable.^25^

Segmentation of the chiasm on CT scans is inherently challenging owing to the absence of HU contrast. Although the DSC scores were very low for this organ, the contours were considered clinically acceptable for photon radiotherapy (Table 6) as the general location of the chiasm was correct. However, for proton therapy, greater accuracy is necessary because of the steeper dose gradients. In general, auto‐segmentation tools performed poorly in delineating head and neck organs. A recent study evaluating the real‐world performance of commercial auto‐segmentation tools found that only 30% of the head and neck contours were clinically acceptable.^30^

On the validation dataset, LimbusAI performed significantly better for the CTV spine of younger patients, as shown in Table 4. LimbusAI did not contour the spinal canal past the spinal cord's inferior border, while the reference labels included the cauda equina. In adults, the spinal cord typically ends at L1‐2, whereas in children, it extends further at around L3. Consequently, when comparing LimbusAI's contours with the reference labels, the dice scores were higher for children because their spinal cord is proportionally longer. Additionally, the lung contours exhibited age‐related trends for LimbusAI for both the validation and test datasets. The model contoured smaller regions on the bottom slices which had a bigger impact on the DSC values for children as their lungs are smaller. Because LimbusAI has not released details of its model and training parameters, the exact reason for underestimating lung contours is unknown. We hypothesize that the contrast windowing used might have been too narrow. A relationship between the DSC and age was also observed for the left kidney. Why this phenomenon occurs in the left kidney and not the right may be attributed to the right kidney's position beneath the liver, facilitating organ detection. In contrast, the left kidney is intricately positioned in proximity to the bowel. With age, there is a greater accumulation of intraabdominal fat, contributing to the enhanced visibility and distinction of the left kidney due to the better visualized fat plane. It is probable that LimbusAI, trained on adult images, emphasizes features associated with the fat plane in the segmentation process.

### 4.2 nnU‐Net

Overall, nnU‐Net demonstrated excellent performance on the validation dataset. Poorer performance was observed for head structures on the test dataset. While nnU‐Net's underlying architecture is based on the simple U‐Net model, its remarkable feature lies in its self‐configuration capability.^22^ The framework automatically selects the most suitable configuration and hyperparameters based on the characteristics of the input images. The training process is fully automated and can be launched with a simple command line.

The model employs substantial data augmentation to enhance robustness and generalizability. One of which is mirroring, which flips the organs horizontally during training. While this approach may be suitable for medical images such as histology slides, it is not suitable for the segmentation of human anatomy. There were many instances where nnU‐Net exhibited difficulty differentiating the left and right eyes, lenses, optic nerves, and cochleae, which resulted in a large variation of DSC values for these structures. In some cases, the left and right kidneys were also reversed (Figure 7). Cases where right and left organs were flipped were considered clinically unacceptable.

Furthermore, the model is designed for generalizability across various medical image modalities which involves a sizable codebase and substantial computation time, necessitating access to high‐performance computers equipped with GPU. Inference of 16 patients in the test dataset required 26.6 GB RAM and 1h 21m run time with a V100 GPU. Despite the hardware requirements, its implementation was straightforward. Data augmentation with mirroring can also be suppressed during training by modifying the code. This was not done in this study as this realization came to light after careful data analysis. One limitation of this study was that we used a 2D model for ease of training and implementation, but the 3D option was also available.

### 4.3 In‐House Models

Given the resource‐intensive and time‐consuming training process of the nnU‐Net, we opted for an in‐house approach with three variants of the U‐Net. This approach also offered the advantage of being tailored to our specific needs while ensuring versatility and scalability. Although training a single end‐to‐end model to accurately contour all structures may have seemed appealing, multiclass image segmentation presented significant challenges in achieving optimal performance. These challenges included the difficulty with input image normalization, class imbalance, and handling large input image size for delineating small structures. While different strategies exist to mitigate these challenges, we opted to leverage domain expertise in radiation oncology and drew inspiration from the clinical workflow. By splitting the task into two stages and training multiple smaller models, the training process became less resource intensive. Additionally, this framework could be scaled down for cases where auto‐segmentation of only a subset of organs is required.

We reframed our first stage as an instance detection task and chose to use YOLOv5 for its ease of implementation.^21^ Various object detection models such as RCNN^31^ and DETR^32^ could also have been used. Note that the key principle in our approach was to remain model agnostic, thus any instance detection algorithm was likely to work well.

This two‐stage approach was not unique. The Ua‐Net described in Chen et al. to segment the head and neck region employed a two‐stage approach, whereby a 3D U‐Net was first used to detect the bounding box containing the OAR of interest, followed by segmentation of the mask.^24^, ^33^ The cascade model in the nnU‐Net also used a similar approach.^15^ What was novel in our method was the leveraging of DRR. Integrating the bounding box of orthogonal 2D projections to reconstruct the 3D ROI led to substantial improvement in both the training and inference times, as well as increased efficiency in computational resources. Detecting OAR instances with YOLOv5 from the DRR of whole‐body CT scans and generating the mini‐CT scans for each organ of interest for the test set of 16 patients took 9 min on a CPU (Intel i5 2.9Ghz with 12GB RAM). Inference took 38 min for the 2D and 2.5D U‐Net, and 46 min for the attention U‐Net.

This bore similarity to the 2.5D U‐Net model proposed by Angermann et al.^34^ This should not be confused with the model described in Chen et al., also called 2.5D U‐Net and as implemented in our in‐house approach. In Angermann et al.’s study, the authors demonstrated the benefits of applying a 2D U‐Net model on orthogonal (coronal and sagittal) images rather than employing a 3D U‐Net directly to recover 3D segmentation masks. Incorporating this method in the second stage of our in‐house approach might have enhanced performance, especially for structures such as the lenses, the kidneys, and the brainstem, where distinguishing the superior‐inferior extent was found to be challenging, resulting in underperformance. Our 2.5D approach, which injected contextual information from slices above and below the slice of interest being contoured mimicked the common practice of scrolling through image stack during organ delineation by radiation oncologists, helped marginally with the performance but not substantially enough. Incorporating attention modules also did not improve the performance appreciably.

### 4.4 Which models to use

Various factors must be considered when introducing a new auto‐segmentation tool into the clinic, including cost, ease of implementation, qualitative expert assessment, and quantitative geometric and dosimetric parameters.^35^ NRG Oncology had formed a working group to establish consensus recommendations for the clinical utilization of AI auto‐segmentation tools.^36^ They recommended that commissioning of such models should include extensive testing across appropriate image modalities, disease sites and clinical scenarios. Detailed documentation of inaccurate performance is also necessary. This work highlighted the limitations of different auto‐segmentation methods for the scenario of pediatric CSI. The trade‐off discussed in the text is summarized in Table 7.

**TABLE 7:** Comparison of different deep learning‐based automatic segmentation approaches.

Approach	Strengths	Limitation
Commercial	• Extensively validated • Generalizable • Easy to use (no AI expertise required)	• Cannot be tailored to specific institutional needs • Not easily adaptable to evolving institutional standards
Out‐of‐the‐box	• Easy to use (minimal AI expertise required) • Can be tailored to specific institutional needs and data • Can be rapidly adapted to evolving institutional standards	• Computationally expensive • Not easily generalizable
In‐house	• Can be tailored to specific institutional needs and data • Can be rapidly adapted to evolving institutional standards • Can be adapted to institutional resources (GPU vs CPU, RAM, etc.)	• AI expertise is required • Not easily generalizable

Abbreviations: AI, artificial intelligence; CPU, central processing unit; GPU, graphics processing unit.

LimbusAI was generally better at generalizing to the external test dataset than the nnU‐Net and in‐house models due to its large and diverse training data. Nevertheless, the performance on both datasets was clinically unsatisfactory for the kidneys and esophagus which were often truncated. Because the details of the model are not available, it is impossible to determine with certainty what caused these errors. This is a major drawback of commercial products. Furthermore, the fact that LimbusAI did not permit the auto‐contouring of the vertebral bodies was problematic in the case of pediatric CSI.

Systematic differences in contouring between institutions cannot be considered when using commercial software such as LimbusAI. Additionally, if an institution wishes to apply auto‐contouring to a patient population that deviates from the norm, such as post‐surgical patients, a model trained with internal data may be better suited. An example of this is online adaptive radiation therapy, where achieving a perfect match between AI‐derived and physician contours for a particular patient is paramount, even if it means sacrificing the reproducibility of results for other patients. In such cases, relying on a model trained with internal data tailored to the specific patient may be more advantageous.

As AI expertise grows within centers, nnU‐Net can be a useful tool as this study has demonstrated that it is easy to implement and showed satisfying results for most structures. However, some modifications might be necessary such as disabling mirroring during data augmentation. If computational resources are limited, we suggest a two‐step approach similar to the in‐house method proposed in this study. Finally, a variational autoencoder (VAE) may be used to obtain more plausible segmentation masks following the method described in Painchaud et al.^37^ In brief, the authors incorporated a constrained VAE into their auto‐segmentation workflow to correct anatomically implausible segmentations of cardiac images produced by a convolutional neural network. VAEs are generative models used to create variations of the input data they were trained on.

## 5 CONCLUSION

We investigated the performance of LimbusAI on pediatric patients and compared it to the nnU‐Net as well as three variants of an in‐house method based on the classic U‐Net. Models trained in‐house can more easily adhere to an institution's standard. Implementing an automated segmentation tool, whether commercial or in‐house, will require careful monitoring by human experts.

## CONFLICT OF INTEREST STATEMENT

The authors declare no conflicts of interest.

## Supporting information



Supporting Information

## References

[1] Vinod SK , Min M , Jameson MG , Holloway LC . A review of interventions to reduce inter‐observer variability in volume delineation in radiation oncology. J Med Imaging Radiat Oncol. 2016;60(3):393‐406. doi:10.1111/1754-9485.12462 27170216

[2] Weber DC , Tomsej M , Melidis C , Hurkmans CW . QA makes a clinical trial stronger: evidence‐based medicine in radiation therapy. Radiother Oncol. 2012;105(1):4‐8. doi:10.1016/j.radonc.2012.08.008 22985777

[3] Vrtovec T , Močnik D , Strojan P , Pernuš F , Ibragimov B . Auto‐segmentation of organs at risk for head and neck radiotherapy planning: from atlas‐based to deep learning methods. Med Phys. 2020;47(9):e929‐e950. doi:10.1002/mp.14320 32510603

[4] Chen W , Wang C , Zhan W , et al. A comparative study of auto‐contouring softwares in delineation of organs at risk in lung cancer and rectal cancer. Sci Rep. 2021;11(1):23002. doi:10.1038/s41598-021-02330-y 34836989 PMC8626498

[5] Adams J , Luca K , Yang X , et al. Plan quality analysis of automated treatment planning workflow with commercial auto‐segmentation tools and clinical knowledge‐based planning models for prostate cancer. Cureus. 2023;15:e41260. doi:10.7759/cureus.41260 37529805 PMC10389787

[6] Urago Y , Okamoto H , Kaneda T , et al. Evaluation of auto‐segmentation accuracy of cloud‐based artificial intelligence and atlas‐based models. Radiat Oncol. 2021;16(1):175. doi:10.1186/s13014-021-01896-1 34503533 PMC8427857

[7] Pera Ó , Martínez Á , Möhler C , et al. Clinical validation of siemens’ syngo.via automatic contouring system. Adv Radiat Oncol. 2023;8(3):101177. doi:10.1016/j.adro.2023.101177 36865668 PMC9972393

[8] Radici L , Ferrario S , Borca VC , et al. Implementation of a commercial deep learning‐based auto segmentation software in radiotherapy: evaluation of effectiveness and impact on workflow. Life. 2022;12(12):2088. doi:10.3390/life12122088 36556455 PMC9782080

[9] Wong J , Fong A , McVicar N , et al. Comparing deep learning‐based auto‐segmentation of organs at risk and clinical target volumes to expert inter‐observer variability in radiotherapy planning. Radiother Oncol. 2020;144:152‐158. doi:10.1016/j.radonc.2019.10.019 31812930

[10] Wong J , Huang V , Giambattista JA , et al. Training and validation of deep learning‐based auto‐segmentation models for lung stereotactic ablative radiotherapy using retrospective radiotherapy planning contours. Front Oncol. 2021;11:626499. doi:10.3389/fonc.2021.626499 34164335 PMC8215371

[11] D'Aviero A , Re A , Catucci F , et al. Clinical validation of a deep‐learning segmentation software in head and neck: an early analysis in a developing radiation oncology center. Int J Environ Res Public Health. 2022;19(15):9057. doi:10.3390/ijerph19159057 35897425 PMC9329735

[12] Wong J , Huang V , Wells D , et al. Implementation of deep learning‐based auto‐segmentation for radiotherapy planning structures: a workflow study at two cancer centers. Radiat Oncol. 2021;16(1):101. doi:10.1186/s13014-021-01831-4 34103062 PMC8186196

[13] Zabel WJ , Conway JL , Gladwish A , et al. Clinical evaluation of deep learning and atlas‐based auto‐contouring of bladder and rectum for prostate radiation therapy. Pract Radiat Oncol. 2021;11(1):e80‐e89. doi:10.1016/j.prro.2020.05.013 32599279

[14] Limbus AI , Press—The latest news from Limbus AI. Accessed December 14, 2024. https://limbus.ai/press

[15] Isensee F , Petersen J , Klein A , et al. nnU‐Net: Self‐adapting Framework for U‐Net‐Based Medical Image Segmentation.arXiv;2018. Accessed February 4, 2024. http://arxiv.org/abs/1809.10486

[16] Isensee F , Jaeger PF , Kohl SAA , Petersen J , Maier‐Hein KH . Net: a self‐configuring method for deep learning‐based biomedical image segmentation. Nat Methods. 2021;18(2):203‐211. doi:10.1038/s41592-020-01008-z 33288961

[17] Hernandez S , Nguyen C , Parkes J , et al. Automating the treatment planning process for 3D‐conformal pediatric craniospinal irradiation therapy. Pediatr Blood Cancer. 2023;70(3):e30164. doi:10.1002/pbc.30164 36591994

[18] Wong K , Gallant F , Szumacher E . Perceptions of Canadian radiation oncologists, radiation physicists, radiation therapists and radiation trainees about the impact of artificial intelligence in radiation oncology—national survey. J Med Imaging Radiat Sci. 2021;52(1):44‐48. doi:10.1016/j.jmir.2020.11.013 33323332

[19] Scheetz J , Rothschild P , McGuinness M , et al. A survey of clinicians on the use of artificial intelligence in ophthalmology, dermatology, radiology and radiation oncology. Sci Rep. 2021;11(1):5193. doi:10.1038/s41598-021-84698-5 33664367 PMC7933437

[20] Diaz O , Guidi G , Ivashchenko O , Colgan N , Zanca F . Artificial intelligence in the medical physics community: an international survey. Phys Med. 2021;81:141‐146. doi:10.1016/j.ejmp.2020.11.037 33453506

[21] Redmon J , Divvala S , Girshick R , Farhadi A , You only look once: unified, real‐time object detection. In: 2016 IEEE Conference on Computer Vision and Pattern Recognition (CVPR) . IEEE; 2016:779‐788. doi:10.1109/CVPR.2016.91

[22] Ronneberger O , Fischer P , Brox T . U‐Net: convolutional networks for biomedical image segmentation. In: Navab N , Hornegger J , Wells WM , Frangi AF , eds. Medical Image Computing and Computer‐Assisted Intervention—MICCAI 2015. Lecture Notes in Computer Science. Springer International Publishing; 2015:234‐241. doi:10.1007/978-3-319-24574-4_28

[23] Oktay O , Schlemper J , Folgoc LL , et al. Attention U‐Net: learning where to look for the pancreas. In: Computer Vision and Pattern Recognition. arXiv; 2018. Accessed February 4, 2024, http://arxiv.org/abs/1804.03999

[24] Chen X , Sun S , Bai N , et al. A deep learning‐based auto‐segmentation system for organs‐at‐risk on whole‐body computed tomography images for radiation therapy. Radiother Oncol. 2021;160:175‐184. doi:10.1016/j.radonc.2021.04.019 33961914

[25] Baroudi H , Brock KK , Cao W , et al. automated contouring and planning in radiation therapy: what is ‘clinically acceptable’?. Diagnostics. 2023;13(4):667. doi:10.3390/diagnostics13040667 36832155 PMC9955359

[26] Hussain MM , Mahmud I . pyMannKendall: a python package for non parametric Mann Kendall family of trend tests. J Open Source Softw. 2019;4(39):1556. doi:10.21105/joss.01556

[27] Kumar K , Yeo AU , McIntosh L , Kron T , Wheeler G , Franich RD , Deep learning auto‐segmentation network for pediatric computed tomography data sets: can we extrapolate from adults?. Int J Radiat Oncol Biol Phys. 2024;119(4):1297‐1306. doi:10.1016/j.ijrobp.2024.01.201 38246249

[28] Hanna EM , Sargent E , ho HuaC , Merchant TE , Ates O . Performance analysis and knowledge‐based quality assurance of critical organ auto‐segmentation for pediatric craniospinal irradiation. Sci Rep. 2024;14(1):4251. doi:10.1038/s41598-024-55015-7 38378834 PMC11310500

[29] Reinke A , Eisenmann M , Dietlinde M , et al. Common limitations of performance metrics in biomedical image analysis. Proc Med Imaging Deep Learn. Published online 2021. https://openreview.net/forum?id=76X9Mthzv4X

[30] Maes D , Gates EDH , Meyer J , et al. Framework for radiation oncology department‐wide evaluation and implementation of commercial artificial intelligence autocontouring. Pract Radiat Oncol. 2024;14(2):e15058. doi:10.1016/j.prro.2023.10.011 37935308

[31] Girshick R , Donahue J , Darrell T , Malik J . Rich feature hierarchies for accurate object detection and semantic segmentation. In 2014 IEEE Conference on Computer Vision and Pattern Recognition, 580-87. Columbus, OH, USA: IEEE. doi:10.1109/CVPR.2014.81

[32] Carion N , Massa F , Synnaeve G , Usunier N , Kirillov A , Zagoruyko S . End‐to‐End object detection with transformers. In: Vedaldi A , Bischof H , Brox T , Frahm JM , eds. Computer Vision—ECCV 2020. Lecture Notes in Computer Science. Springer International Publishing; 2020:213‐229. doi:10.1007/978-3-030-58452-8_13

[33] Tang H , Chen X , Liu Y , et al. Clinically applicable deep learning framework for organs at risk delineation in CT images. Nat Mach Intell. 2019;1(10):480‐491. doi:10.1038/s42256-019-0099-z

[34] Angermann C , Haltmeier M . Random 2.5D U‐net for Fully 3D Segmentation. In: Liao H , Balocco S , Wang G , eds. Machine Learning and Medical Engineering for Cardiovascular Health and Intravascular Imaging and Computer Assisted Stenting. Lecture Notes in Computer Science. Springer International Publishing; 2019:158‐166. doi:10.1007/978-3-030-33327-0_19

[35] Heilemann G , Buschmann M , Lechner W , et al. Clinical implementation and evaluation of auto‐segmentation tools for multi‐site contouring in radiotherapy. Phys Imaging Radiat Oncol. 2023;28:100515. doi:10.1016/j.phro.2023.100515 38111502 PMC10726238

[36] Rong Y , Chen Q , Fu Y , et al. NRG oncology assessment of artificial intelligence deep learning–based auto‐segmentation for radiation therapy: current developments, clinical considerations, and future directions. Int J Radiat Oncol. 2024;119(1):261‐280. doi:10.1016/j.ijrobp.2023.10.033 PMC11023777 37972715

[37] Painchaud N , Skandarani Y , Judge T , Bernard O , Lalande A , Jodoin PM . Cardiac segmentation with strong anatomical guarantees. IEEE Trans Med Imaging. 2020;39(11):3703‐3713. doi:10.1109/TMI.2020.3003240 32746116

