# Supplementary material for: Trade‐off of different deep learning‐based auto‐segmentation approaches for treatment planning of pediatric craniospinal irradiation autocontouring of OARs for pediatric CSI
Source: Med Phys. 2025 Apr 1;52(6):3541–56. doi: 10.1002/mp.17782 (PMC12149715; doi:10.1002/mp.17782)
Supplement: Supplementary file 1 — Supporting Information [file MP-52-3541-s001.pdf]

Supplemental Tables

OAR name	Coronal	Sagittal
CTV_Spine	Bone	Soft tissue
OTV_VB	Bone	Bone
Esophagus	Lung	Soft tissue
Kidney_L	Composite	Composite
Kidney_R	Soft tissue	Soft tissue
Lung_L	Lung	Lung
Lung_R	Lung	Lung
Brain	Bone	Bone
Brainstem	Bone	Bone

Supplemental Table 1 – The windowing scheme employed for the coronal and sagittal Digitally Reconstructed Radiographs (DRRs) in YOLO for determining the bounding box that encompasses the Organ at Risk (OAR).

OAR name	Maximum axial width	Maximum axial height
CTV_Spine	256	128
OTV_VB	256	128
Esophagus	128	128
Kidney_L	128	128
Kidney_R	128	128
Lung_L	256	256
Lung_R	256	256
Brain	256	256
Brainstem	128	128
Cochlea_L	32	32
Cochlea_R	32	32
Eye_L	64	64
Eye_R	64	64
Lens_L	64	64
Lens_R	64	64
Optic_Nerve_L	128	64

Optic_Nerve_R	128	64
Chiasm	128	128

Supplemental Table 2 – Padding of 0 was used to obtain uniform input image size in pixel for each OAR.

OAR name	Maximum number of epochs	Learning rate
CTV_Spine	600	$1e^{-6}$
OTV_VB	600	$1e^{-6}$
Esophagus	700	$1e^{-6}$
Kidney_L	800	$1e^{-6}$
Kidney_R	800	$1e^{-6}$
Lung_L	600	$1e^{-6}$
Lung_R	600	$1e^{-6}$
Brain	500	$1e^{-6}$
Brainstem	1200	$1e^{-6}$
Cochlea_L	200	$1e^{-4}$
Cochlea_R	200	$1e^{-4}$
Eye_L	200	$1e^{-4}$
Eye_R	200	$1e^{-4}$
Lens_L	200	$1e^{-4}$
Lens_R	200	$1e^{-4}$
Optic_Nerve_L	200	$1e^{-4}$
Optic_Nerve_R	200	$1e^{-4}$
Chiasm	1000	$1e^{-4}$

Supplemental Table 3 – Training epochs and learning rates used for training the U-Net. All variants used the same parameters. The AdamW optimizer was used.

Supplemental Figures

A)

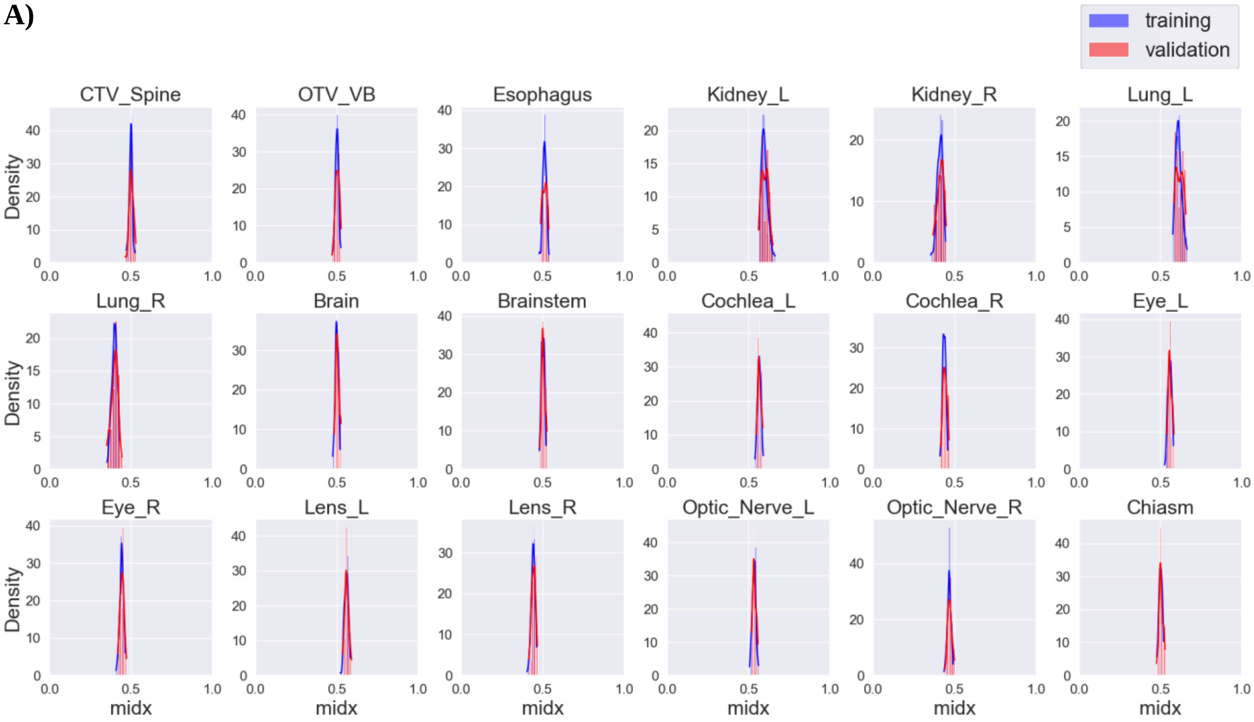

B)

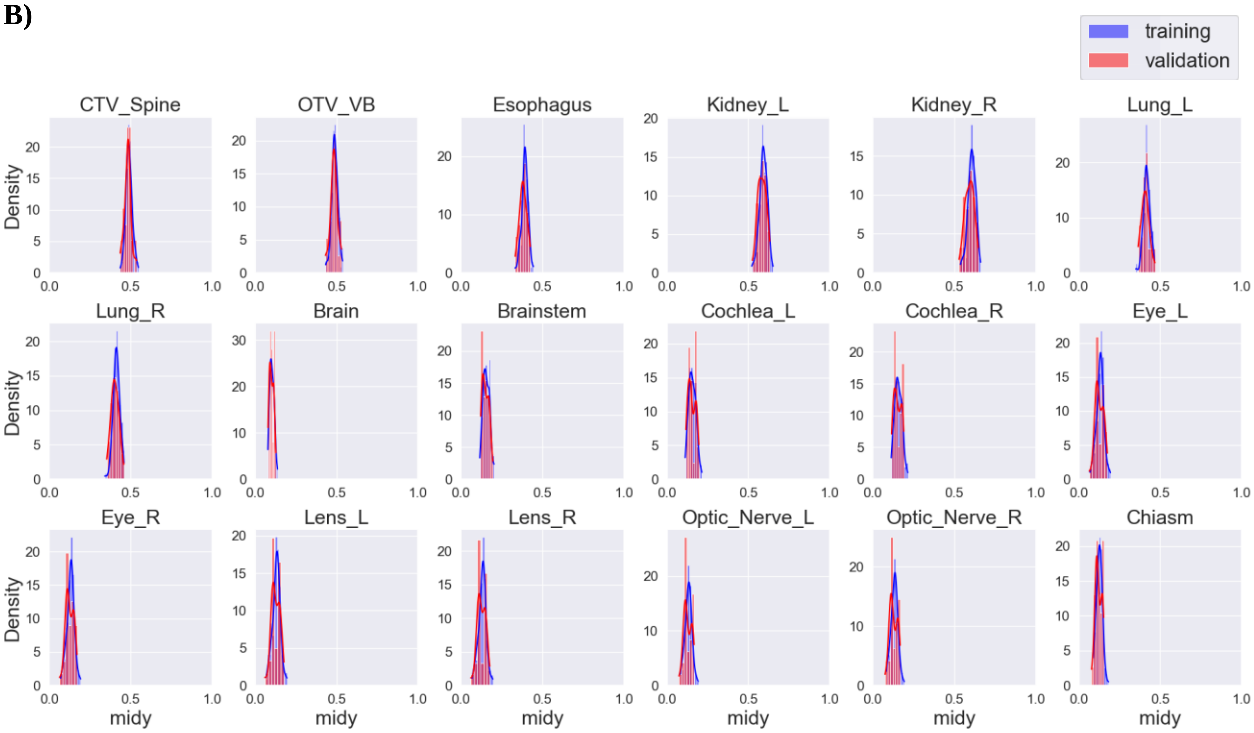

C)

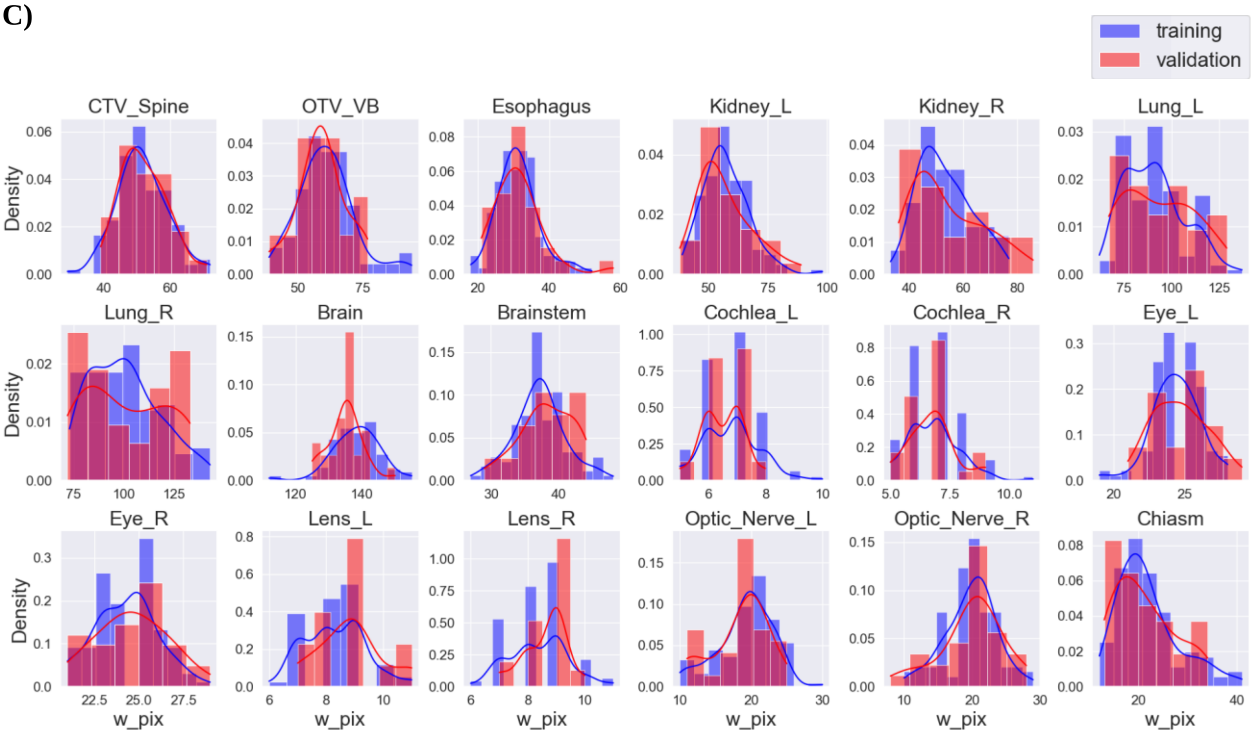

D)

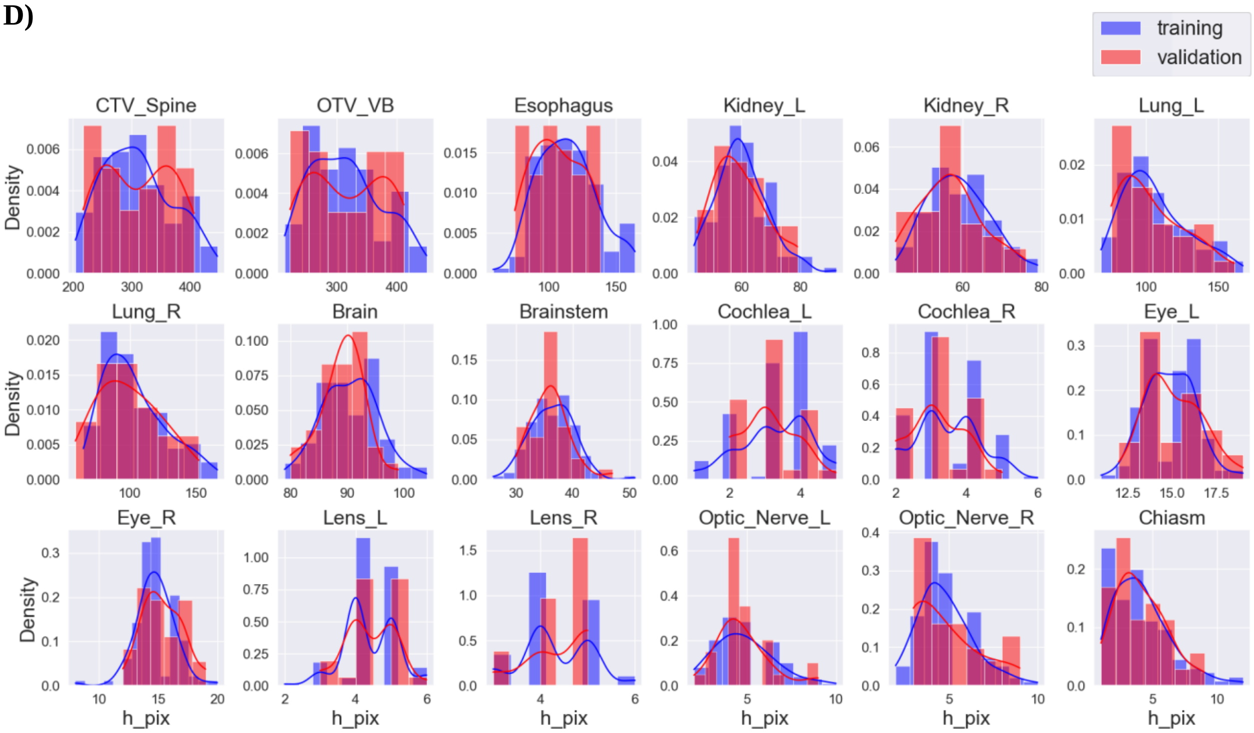

Supplemental Figure 1 – To verify that the training and validation datasets were balanced in terms of organ dimensions, orthogonal bounding boxes were created around each OAR in the coronal and sagittal

planes. These bounding boxes are defined by their mid-point coordinates (x, y), as well as by their width and height (w, h). The values x and w were normalized to the number of slices in the anterior-posterior direction (512), while y and h were normalized to the number of slices in the superior-inferior direction (patient-dependent, always starts one slice above the scalp vertex and ends just below the ischium). The figure shows the distribution of these variables for every organ and confirms that they are well balanced between datasets. The distribution of the normalized mid-point (x, y) coordinates in the coronal (A) and the sagittal (B) planes, as well as the distribution of the width and height of the bounding boxes in the coronal (C) and sagittal (D) planes. Blue bars refer to the training dataset while the red bars are the validation dataset.

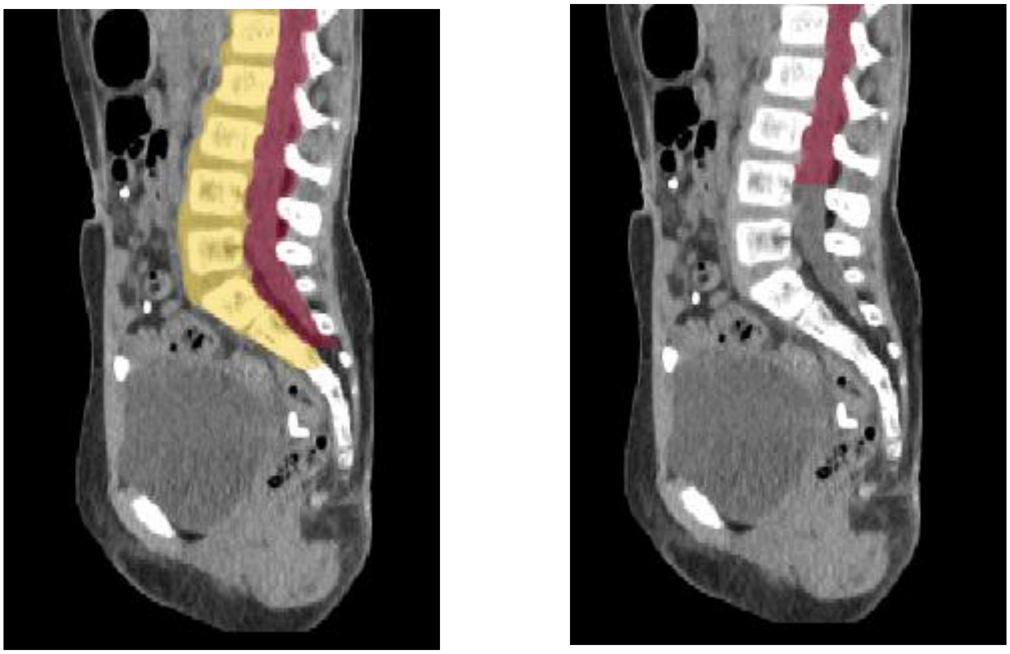

Supplemental Figure 2 – Sagittal slice of a patient showing the CTV spine (red) and OTV VB (yellow). The left panel shows the reference contours and the right panel shows the contours created by LimbusAI.

Architecture of In-House Models

Supplemental Figure 3 illustrates how our implementation of the basic 2D U-Net differed from the classic architecture. Specifically, the output of the first level was 32 channels instead of 64. Each subsequent level increased the number of channels by a factor of 2. We used max pooling with a kernel size of 2 x 2 and a stride of 2 to downsample between each level in the encoder. The input to the bottleneck layer was 256 channels while its output was 512 channels. For the decoder, we used 2D transposed convolution with a kernel size of 2 x 2 and a stride of 2 for upsampling between levels. Within each level, we used two convolution layers. Each convolution layer consisted of a 3 x 3 kernel with a padding of 1 without bias. ReLU was used as the nonlinear function after each convolutional layer with a dropout of 0.15 during training. The hyperparameters are detailed in Supplemental Table 3. Supplemental Figure 4 illustrates the attention U-Net, which included attention gates in the skip connections between the encoder and decoder to highlight the most relevant areas of the input image. Supplemental Figure 5 shows the 2.5D U-Net architecture, which took in a three-channel image as input, corresponding to the target axial slice, sandwiched between the superior and inferior axial slices. The model’s output was the segmentation mask specifically for the middle axial slice.

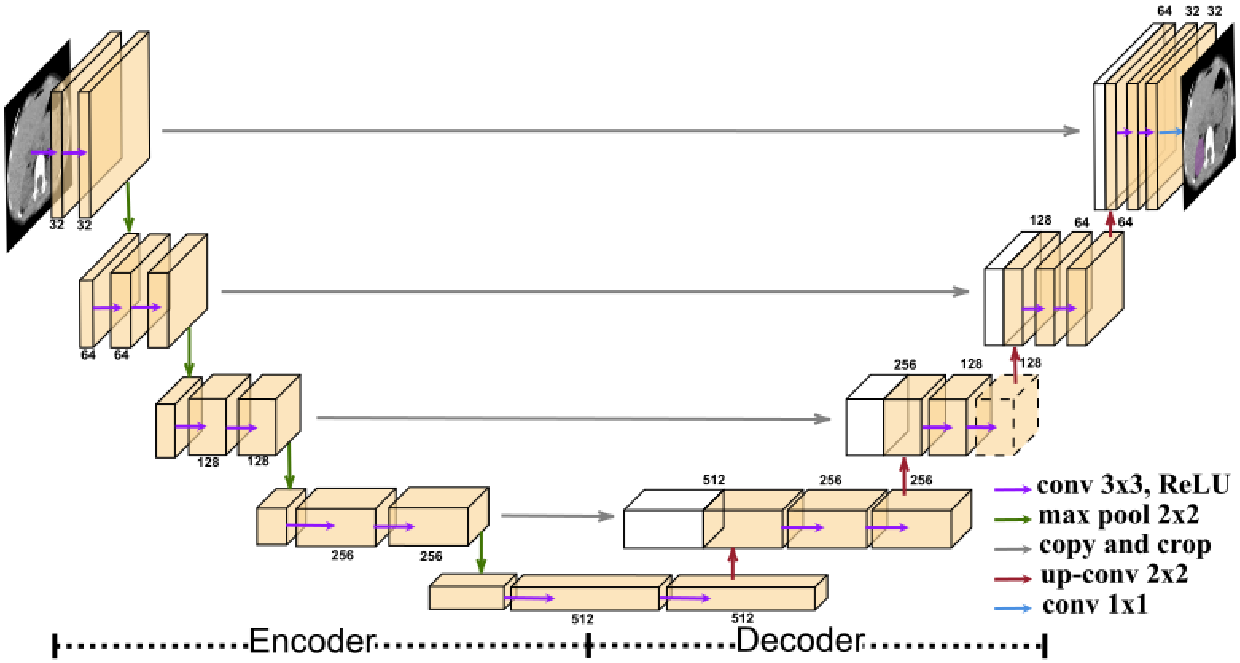

Supplemental Figure 3 – 2D U-Net architecture.

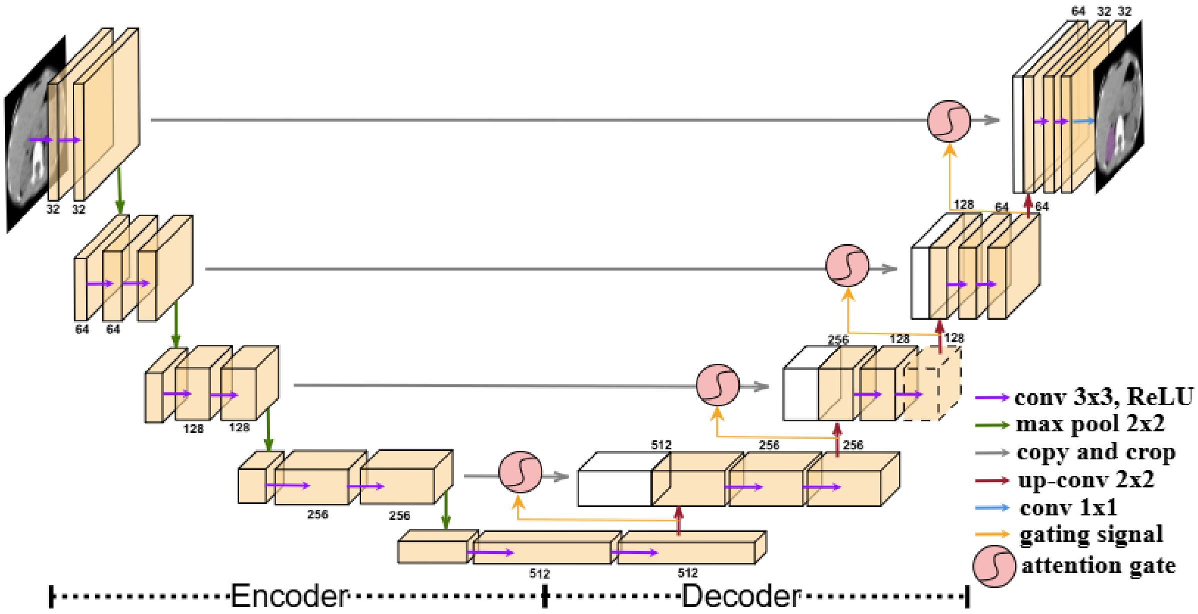

Supplemental Figure 4 – Attention U-Net architecture.

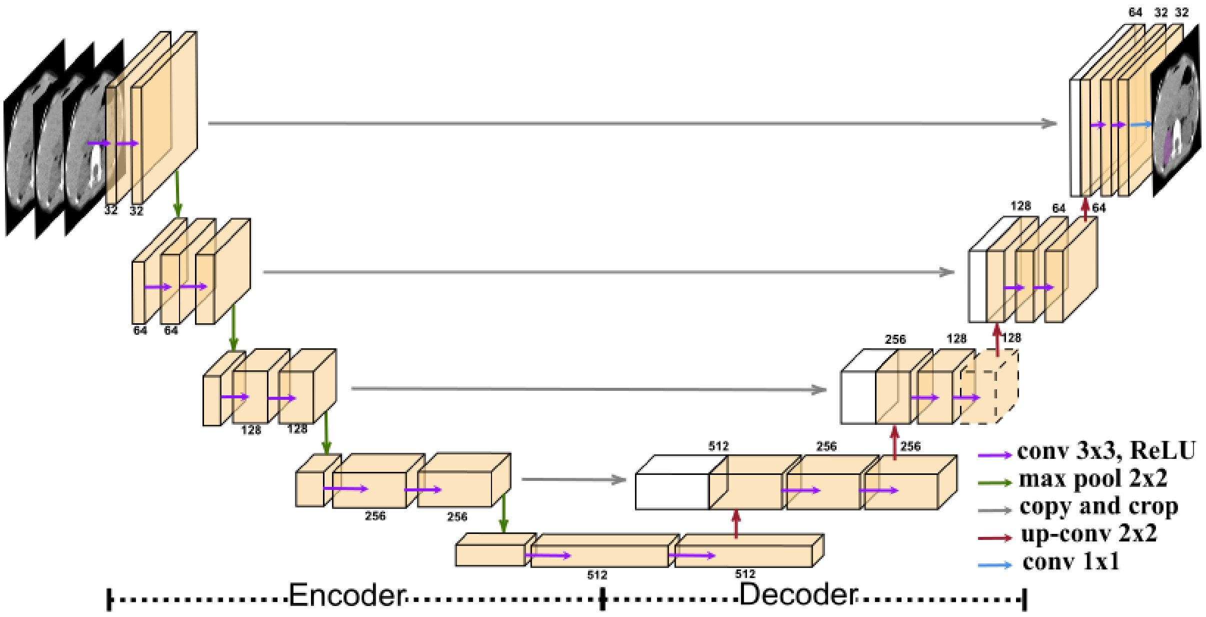

Supplemental Figure 5 – 2.5D U-Net architecture.

Clinical Acceptability Analysis

Supplemental Figures 6 and 7 show the breakdown of clinical acceptability results for the validation and test datasets, respectively. A five-point Likert scale was used to score every contour. A score of 4 or higher is considered clinically acceptable.

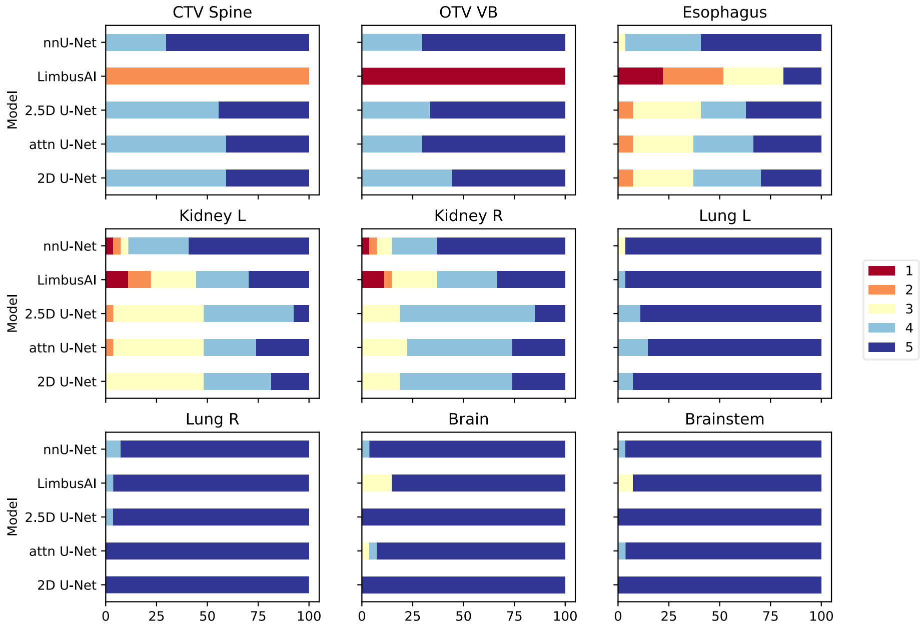

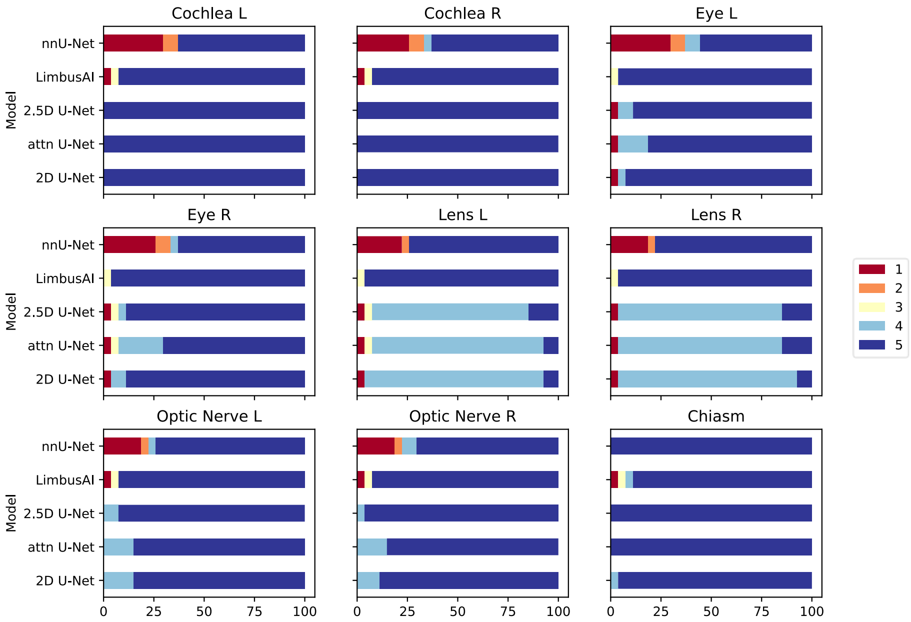

Supplemental Figure 6 – Clinical acceptability results of contours on the validation dataset determined using a five-point Likert scale.

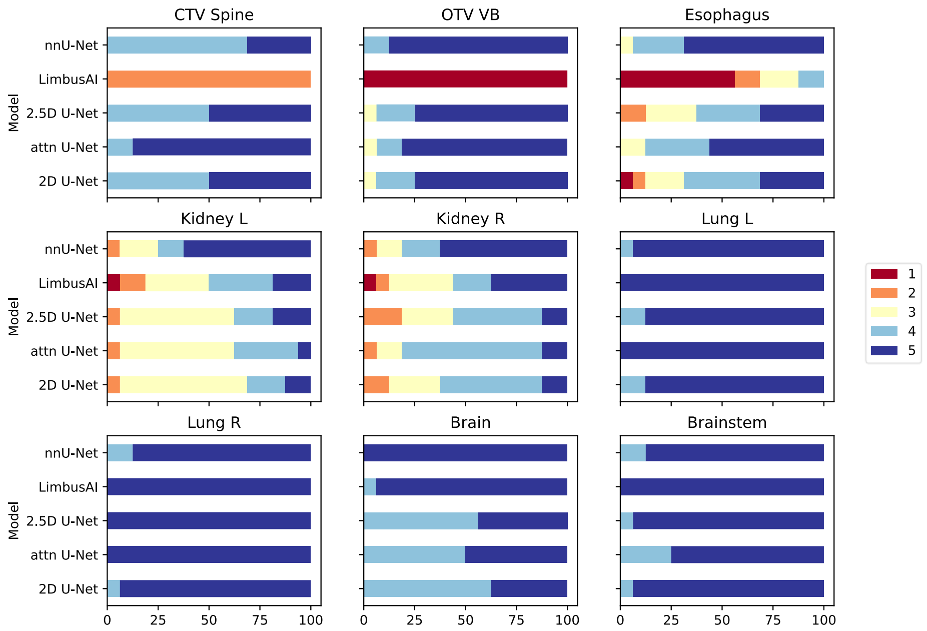

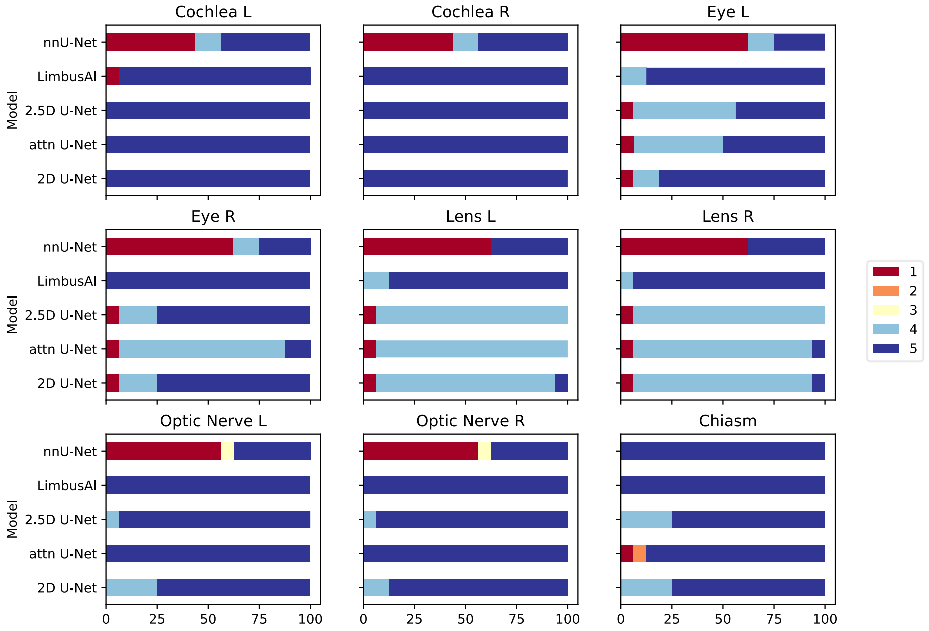

Supplemental Figure 7 – Clinical acceptability results of contours on the test dataset determined using a 5-point Likert scale.
